# Shotgun metagenomic mining reveals a new FAD-dependent D-lactate dehydrogenase in an isopod gut microbiome

**DOI:** 10.1128/aem.01480-25

**Published:** 2025-11-13

**Authors:** Catarina Coelho, André Taborda, Constança Lorena, Tomás Frazão, António Veríssimo, Patrícia T. Borges, Vânia Brissos, Igor Tiago, Lígia O. Martins

**Affiliations:** 1 Instituto de Tecnologia Química e Biológica António Xavier, Universidade Nova de Lisboa, Oeiras, Portugal; 2 Centro de Ecologia Funcional, Departamento Ciências da Vida, Universidade de Coimbra 37829, Coimbra, Portugal; Danmarks Tekniske Universitet The Novo Nordisk Foundation Center for Biosustainability, Kgs. Lyngby, Denmark

**Keywords:** metagenomics, oxidoreductases, structural biology, biocatalysis, pyruvate production

## Abstract

Shotgun metagenomic sequencing has emerged as a powerful tool for exploring microbial diversity and uncovering genes encoding novel biocatalysts from complex environments. Here, we report the discovery and characterization of a new FAD-dependent D-lactate dehydrogenase (PdG-D-LDH) from the gut microbiome of the isopod *Porcellio dilatatus*. The enzyme was identified through *in silico* screening using BLAST and AlphaFold3 and functionally characterized as a homodimeric, thermoactive, and thermostable protein, demonstrating the robustness required for biotechnological applications. PdG-D-LDH exhibits a strong catalytic preference toward D-lactate and preferentially reduces quinones over cytochrome *c* or molecular oxygen. X-ray crystallography revealed a VAO/PCMH-like fold with a solvent-accessible active site that harbors both a FAD cofactor and an Fe(II) ion. Molecular docking studies provided insights into the structural determinants of its stereoselective substrate recognition. Under mild conditions, the enzyme catalyzed the oxidation of D-lactate to pyruvate with a 90% yield after 24 h of reaction, using molecular oxygen as the electron acceptor.

**IMPORTANCE:**

This study illustrates how metagenomics, structural biology, and computational tools can jointly drive the discovery of new enzymes with valuable biotechnological applications aligned with circular economic principles. The newly identified D-lactate dehydrogenase, PdG-D-LDH, exhibits thermostability, stereoselectivity, and high catalytic efficiency, providing new insights into the structure-function relationships of lactate-metabolizing enzymes.

## INTRODUCTION

Lactic acid, or lactate, is one of the most abundant α-hydroxy acids in nature and plays a central role in cellular metabolism. It is widely used in food, cosmetics, pharmaceutical, and chemical industries, notably as a precursor for polylactic acid (PLA), a key biodegradable and biocompatible biopolymer (1). Enzymatic production of lactate through sugar fermentation from lignocellulosic biomass, such as agricultural residues and forest waste, offers a sustainable and attractive biosynthetic route, with the added advantage of enantiomeric selectivity depending on the lactic acid bacteria used (2, 3). Lactate exists as two enantiomers due to its chiral C2 atom. The production of D- and L-lactate from pyruvate is catalyzed by NAD-dependent lactate dehydrogenases (LDHs) during anaerobic fermentation. Notably, polylactic acid (PLA) derived from D-lactate exhibits enhanced mechanical strength and thermal stability. Consequently, various lactic acid bacterial strains have been investigated for the cost-effective production of optically pure D-lactate (4–7). On the other hand, both lactate enantiomers are primarily catabolized to pyruvate by enzymes that catalyze the reverse reaction, which are flavin-dependent LDHs, often referred to as respiratory or NAD-independent LDHs (8–11); some, such as L-lactate oxidases (L-LOX), even utilize molecular oxygen as an electron acceptor (12). Pyruvate and its derivatives (e.g., acetoin, butanol, butyrate, butanediol, and alanine) have a growing demand in the pharmaceutical, cosmetic, and food sectors (13, 14), but suffer from traditional chemical synthesis involving harsh conditions, toxic reagents, and significant energy consumption (15). Biocatalytic approaches provide an attractive alternative, offering simpler reaction mixtures, high substrate conversion, and more straightforward product recovery compared to fermentation.

Over the past decade, an increasing number of lactate-oxidoreductase enzymes (L- and D-LDHs and L-LOXs) have been reported, significantly advancing our understanding of lactate metabolism and utilization (16–18). However, only eight X-ray crystal structures have been deposited in the Protein Data Bank (PDB), highlighting significant gaps in our understanding of the structural and mechanistic properties of this class of enzymes, despite their promising biotechnological potential. In this study, we address this limitation by investigating a new FAD-dependent D-LDH, a subgroup of lactate-oxidoreductases that remain comparatively underexplored. Building on our recent gut metagenomic analysis of the soil-feeding invertebrate *Porcellio dilatatus* (19), we employed an integrated approach combining next-generation sequencing, sequence-based comparative analysis, and state-of-the-art deep learning structure prediction tools, including AlphaFold3 (20) and RoseTTAFold (21), to identify an enzyme here designated PdG-D-LDH. We performed a comprehensive functional and structural characterization of this enzyme, using phylogenetic, biochemical, kinetic, and computational analyses. Our findings not only expand the current repertoire of known D-LDHs but also provide new insights into their structure–function relationships, contributing to the broader understanding of lactate metabolism and supporting their further development for sustainable biotechnological applications. This study identified an interesting enzyme that catalyzes the oxidation of D-lactate to pyruvate, providing a valuable basis for biocatalyst and bioprocess investigations.

## RESULTS AND DISCUSSION

### Metagenomic DNA assembly, microbial taxonomy, and functional annotation

After a brief analysis of the structural diversity of microbial communities, the genomic DNA from six *P. dilatatus* gut samples was pooled and subdivided into three aliquots, which were sequenced in parallel using an Illumina sequencing platform. Shotgun metagenomic sequencing was performed, and after quality control, 93,710,998 high-quality paired-end reads were obtained. A *de novo* assembly operating in metagenome mode recognized 29,215 assembled contigs, totaling 49,584,674 bp, with an N50 of 1,645 bp and a GC content of 59.59%. Contigs were used to generate protein sequences, and 63,409 protein sequences were obtained and analyzed after quality control.

Taxonomic composition analysis revealed that most sequences were classified as belonging to the domain Bacteria (59%). Only a minor fraction (1%) was assigned as viruses, and 2% were designated eukaryotic cells associated with arthropods. The remaining 38% of the sequences were assigned as unclassified (“undefined” or “others”), indicating that these sequences were not identified in the reference database. The bacterial populations were distributed among the phyla *Actinomycetota* (55%) and *Pseudomonadota* (4%), with *Alphaproteobacteria* (3%) and *Gammaproteobacteria* (1%) being the dominant classes within the phylum *Pseudomonadota*.

**Fig 1:**
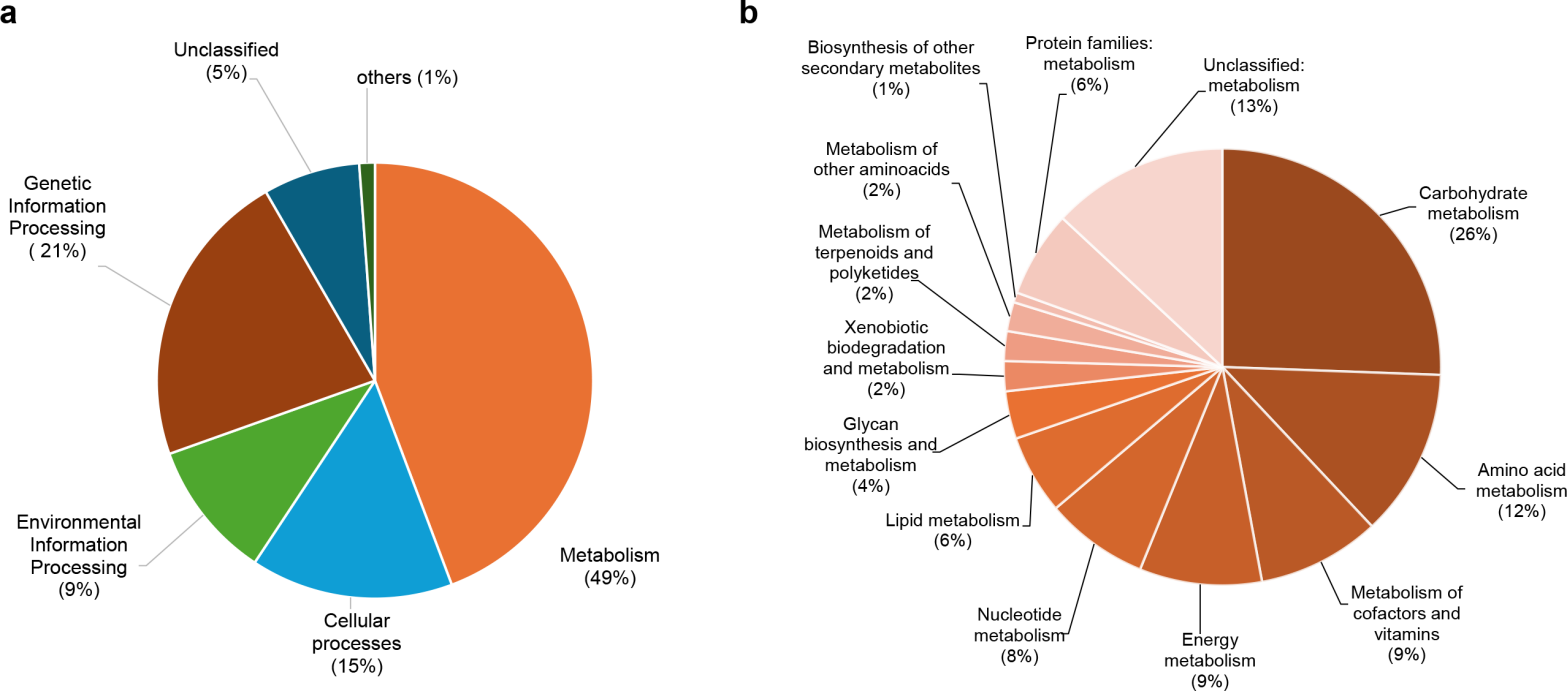
Functional diversity determined by the *P. dilatatus* gut microbiome. (**a**) All functional modules and (**b**) metabolisms within the “metabolism” functional module.

The functional analysis of the sequences (Fig. 1) revealed that nearly half (49%) of the identified sequences were linked to the “metabolism functional module.” Within this module, “carbohydrate metabolism” was the most abundant (26%), followed by “amino acid metabolism” (12%), “energy metabolism” (9%), “metabolism of cofactors and vitamins” (9%), and “lipid metabolism” (9%). These findings align with the functional predictions made in (19). The emphasis on metabolic processes, particularly “carbohydrate metabolism,” is concordant with other reports (22), indicating that gut microbiomes are rich in carbohydrate-metabolizing enzymes.

### Identification of a new D-lactate dehydrogenase by metagenomic mining

The gut microbiome of the terrestrial isopod *P. dilatatus* provides a model for studying microbial enzymes adapted to specialized niches, where lactate metabolism may support microbial community stability and host–microbe interactions (19). D-LDHs catalyze the oxidation of D-lactate to pyruvate and play key metabolic roles. While NAD-dependent L-LDHs are well studied, FAD-dependent D-LDHs remain poorly characterized, particularly in bacteria, highlighting the need for further investigation of their catalytic diversity and physiological functions (23, 24). The shotgun metagenome data obtained from the *Porcellio dilatatus* gut microbiome (PdG.Database) were used as a source to identify a new D-LDH. The two D-lactate dehydrogenases with available biochemical and structural data, *Mus musculus* (PDB 8JDE (23) and *Acetobacterium woodii* (PDB 7QH2, chain C) (25), were used as queries in a standalone BLAST search. The resulting hits were ranked by sequence similarity (Table S1). This latter structure is derived from a high-resolution cryo-EM structure of a lactate dehydrogenase flavoprotein in complex with two electron-transfer proteins, specifically the complex LDH-EtfAB, which is a member of a family that contains a dehydrogenase/oxidoreductase core and several peripherally attached EtfAB modules. This analysis resulted in the identification of a predicted protein sequence (NODE_10957) with 465 amino acids that shared 33.7% and 32.2% identity with PDBs 7QH2 and 8JDE, respectively. This protein sequence, named PdG-D-LDH, was selected for further biochemical and enzymatic characterization. A preliminary structural analysis based on the AlphaFold3-predicted model revealed the highest degree of structural similarity to the D-LDH from *M. musculus* (PDB 8JDE), showing 1.86 Å RMSD value between the two proteins (Fig. S1). In terms of amino acid sequence similarity with previously deposited proteins, PdG D-LDH exhibited high homology to FAD-binding oxidoreductases from diverse *Nocardiopsis* species (Table S2), suggesting a conserved evolutionary relationship within this extremophilic genus (26).

### Phylogenetic analysis of PdG-D-LDH

**Fig 2:**
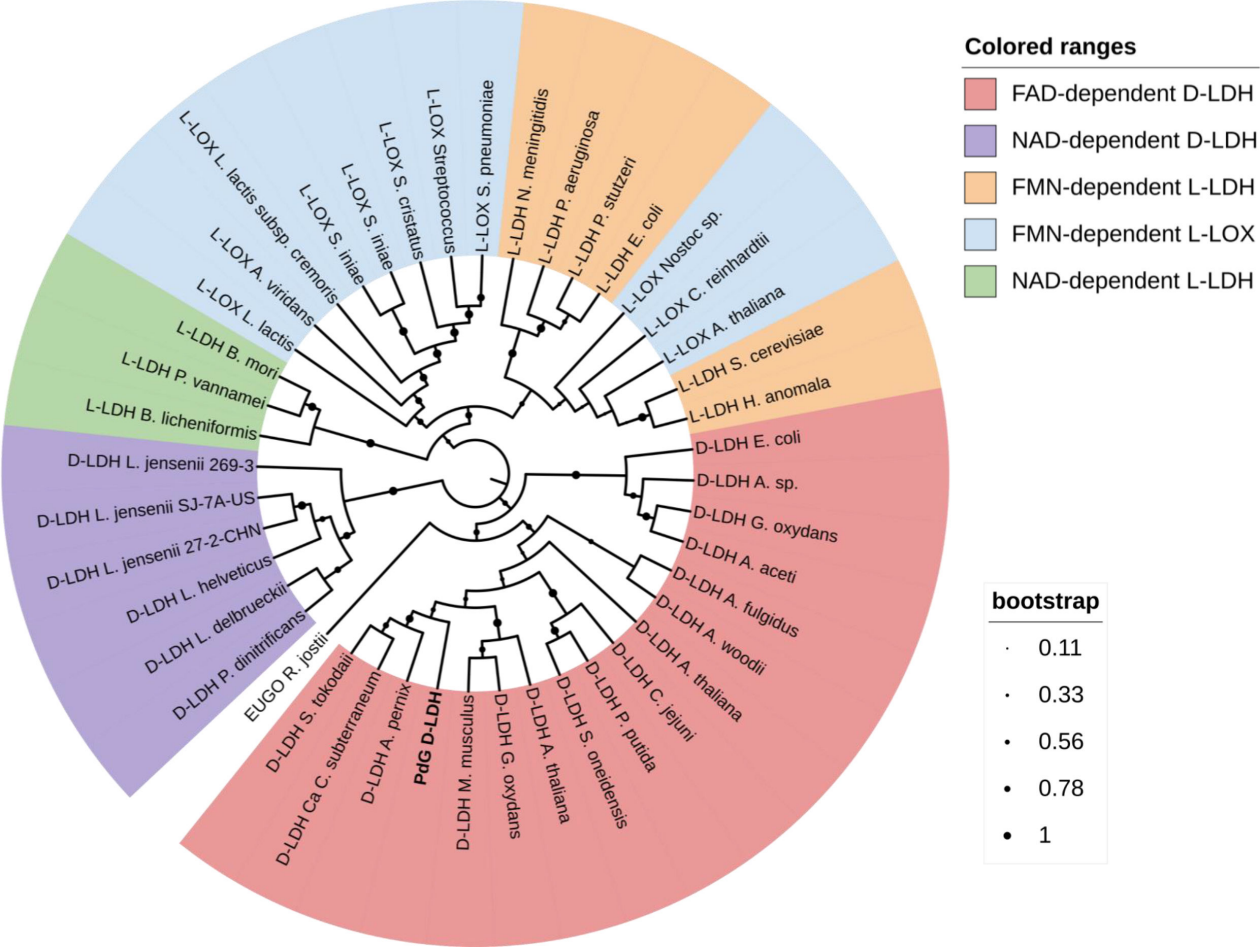
Sequence similarity tree showing the relationship between PdG-D-LDH, LOX, and LDH enzymes. PdG-D-LDH and their lactate oxidases and lactate dehydrogenases have been experimentally characterized or solely annotated in online databases. L-LOX, L-lactate oxidase; D-LDH, D-lactate dehydrogenase; L-LDH, L-lactate dehydrogenase; EUGO, eugenol oxidase. The phylogenetic tree was constructed using the maximum likelihood method with 500 bootstraps in MEGA11. The D-LDH characterized in this work is in bold. In the phylogenetic tree, the D-lactate dehydrogenases are divided into different clusters: NAD-dependent D-LDH (purple), FAD-dependent D-LDH (red), NAD-dependent L-LDH (green), FMN-dependent L-LDH (orange), and FMN-dependent L-LOX (blue). The two amino acid sequences of the nearest PDBs (7QH2 and 8JDE) were also included in the analysis. LDH and LOX enzymes from different origins were selected for this analysis. From the archaeal origin, the D-LDH from *Aeropyrum pernix, Archaeoglobus fulgidus, Candidatus Caldiarchaeum subterraneum*, *Thermoproteus tenax,* and *Sulfolobus tokodaii*. From bacterial origin, *Acetobacter aceti*, *Acetobacterium woodii*, *Acidocella* sp., *Aerococcus viridians*, *Bacillus licheniformis*, *Campylobacter jejuni*, *Escherichia coli*, *Gluconobacter oxydans*, *Pseudomonas stutzeri*, *Pseudomonas aeruginosa*, *Pseudomonas putida*, *Paracoccus denitrificans*, *Lactobacillus jensenii*, *Lactobacillus delbrueckii*, *Lactobacillus lactis*, *Lactobacillus helveticus*, *Neisseria meningitidis*, *Shewanella oneidensis*, *Streptococcus cristatus*, *Streptococcus pneumoniae,* and *Streptococcus iniae*. From eukarya origin, *Arabidopsis thaliana, Bombyx mori*, *Chlamydomonas reinhardtii*, *Mus musculus*, *Hansenula anomala*, *Penaeus vannamei*, *Saccharomyces cerevisiae*, and *Nostoc* sp. The eugenol oxidase from *Rhodococcus jostii* (35) was used as an outgroup. The phylogenetic tree was drawn using iTol (36).

A phylogenetic analysis was performed to evaluate the sequence similarity of the PdG-D-LDH with lactate oxidases (LOX) and L- and D-lactate dehydrogenases (LDH) reported in the literature (Fig. 2; Table S3). LDHs can be either NAD-dependent or flavin-dependent. NAD-dependent LDHs feature a Rossmann fold for NAD(H) binding (27). NAD-dependent D-LDHs share the same fold as the NAD-dependent L-LDHs and are part of the D-isomer-specific 2-hydroxyacid dehydrogenase family (28). These enzymes are integral to cellular energy metabolism and are tightly regulated by the intracellular redox NAD^+^/NADH ratio, which under physiological conditions favors lactate formation. Flavin-dependent L-LDHs (PDB 1FCB) (also known as NAD-independent L-LDHs) and L-LOXs (PDBs 2DU2 and 6RHT) belong to the FMN-dependent α-hydroxyacid dehydrogenase family and share a TIM barrel (α/β barrel) fold (12, 29–34). In contrast, flavin-dependent D-LDHs belong to the FAD-binding oxidoreductase/transferase type 4 family, exhibiting a characteristic vanillyl alcohol oxidase/para-cresol methylhydroxylase (VAO/PCMH) (PDBs 8JDE, 7QH2, 1F0X) structural fold (12, 30, 31). The PdG-D-LDH sequence is grouped within FAD-dependent D-LDH sequences (Fig. 2, red). It is positioned within a clade that is composed of enzyme members from archaeal sources, suggesting that PdG-D-LDH shares a closer evolutionary lineage with archaeal enzymes.

### Purification and cofactor identification of PdG-D-LDH

The recombinant protein PdG-D-LDH was heterologously produced in *Escherichia coli*. The enzyme was recovered from the soluble fraction, which suggests that, unlike other D-LDHs (9), it is a cytoplasmic enzyme. Further supporting this, critical amino acid residues known to be part of the membrane-binding domain are not conserved within its sequence, as found, for example, in D-LDHs from *E. coli* (8), where the protein is located on the cytoplasmic side of the inner membrane and is involved in electron transfer. PdG-D-LDH was purified using a two-step purification using affinity chromatography followed by size exclusion. The protein was identified as a band at approximately 50 kDa on an SDS-PAGE (Fig. S2a), in agreement with the molecular weight estimated from the gene sequence (49.8 kDa). However, in size exclusion chromatography, approximately 38% of the enzyme eluted in a peak corresponding to 439 ± 18 kDa, and approximately 64% of the enzyme eluted in a peak corresponding to 97 ± 2 kDa (Fig. S2b). This result suggests that PdG-D-LDH exists in solution as a mixture of oligomeric states; however, only the dimeric fraction (97 kDa) exhibits a yellowish color and shows enzymatic activity (Fig. S2c and d). Indeed, VAO octamerization is mediated by a specific dimer–dimer interaction loop (residues 220–235), whose deletion converts the enzyme from octamers to dimers without affecting activity (37). Structural comparison shows that PdG-D-LDH lacks the corresponding loop, consistent with its dimeric state. It would be interesting in future studies to introduce this loop into PdG-D-LDH to test whether it promotes oligomerization. In VAO, FAD binding acts as a trigger for assembly into octamers (38, 39). By contrast, PdG-D-LDH remains a dimer despite containing FAD, indicating that the cofactor does not drive oligomerization in this enzyme. This dimeric purified fraction exhibits a UV-vis absorption spectrum with maxima at 390 nm and 450 nm (Fig. S3a), characteristic of flavoproteins. HPLC analysis of the flavin released from the pure enzyme preparation shows a retention time of 4.41 min, identical to that of the flavin adenine dinucleotide (FAD) standard (4.29 min), confirming that FAD is the cofactor present in PdG-D-LDH (Fig. S3b). Unlike many other members of the VAO/PCMH family (31, 40), the FAD cofactor is not covalently attached to the enzyme, as it was released upon chemical treatment with SDS (Fig. S3a). Beyond these typical flavin peaks, the UV-Vis spectrum displays a band at approximately 340 nm, which may reflect contributions from an iron ion in the vicinity of the FAD cofactor (36). This was confirmed by ICP-OES analysis, which revealed a ratio of 0.7 mol of iron per mol of the enzyme. The enzyme production yields 14 ± 5 mg of total enzyme per liter of *E. coli* culture and contains a flavin content of 28 ± 9%. Attempts to increase enzyme flavinylation *in vitro* were unsuccessful. All experimental work was performed using this purified functional fraction.

### Electron acceptor and donor substrate specificity of PdG-D-LDH

The enzyme’s ability to reduce various electron acceptors was assessed by UV-Vis, monitoring flavin re-oxidation after reduction with dithionite. We used the artificial electron acceptor 2,6-dichlorophenolindophenol (DCIP), as well as molecular oxygen, NAD(P)^+^, and various quinones: anthraquinone, menaquinone, lawsone, 1,2-naphthoquinone-4-sulfonic acid (NQS), 1,4-naphthoquinone, menadione, and 1,4-benzoquinone (Fig. S4). Flavin oxidation by PdG-D-LDH occurred fastest with DCIP, followed by quinones and then oxygen, indicating that the enzyme functions more efficiently as a dehydrogenase than as an oxidase. NAD^+^ and NADP^+^ are not substrates. To assess electron donors, assays were conducted using various acids, alcohols, sugars, and aldehydes as electron donors, with DCIP as the acceptor. The highest activity was observed with D-lactate (100%), followed by L-lactate (33%), with only residual activity for other substrates (Table 1). L-lactate was used in several biochemical assays due to its considerably lower cost.

**TABLE 1:** Substrate scope of purified PdG-D-LDH^*a*^

Substrate (electron donor)	Relative activity (%)
α-Hydroxy acids	
D-lactate	100
L-lactate	33
D, L-malic acid	4.2
Succinic acid	2.4
Mandelic acid	2.0
Maleic acid	1.4
Aldehydes	
Furfural aldehyde	2.0
Sugars	
Ribose	1.8
Fructose	1.1
Mannose	0.75
Glucosamine	0.57
Lactose	0.39
Alcohols	
Sinapyl alcohol	0.68
2-Phenylethanol	0.54
4-Aminobenzyl alcohol	0.14

^*a*^ Activities were measured using 10 mM substrate, except for furfural aldehyde (5 mM) with 0.075 mM DCIP in 100 mM sodium phosphate buffer (pH 7.5) at RT. 100% activity corresponds to a Vmax = 1.2 U/mg of protein.

### Dependence of temperature and pH on activity

**Fig 3:**
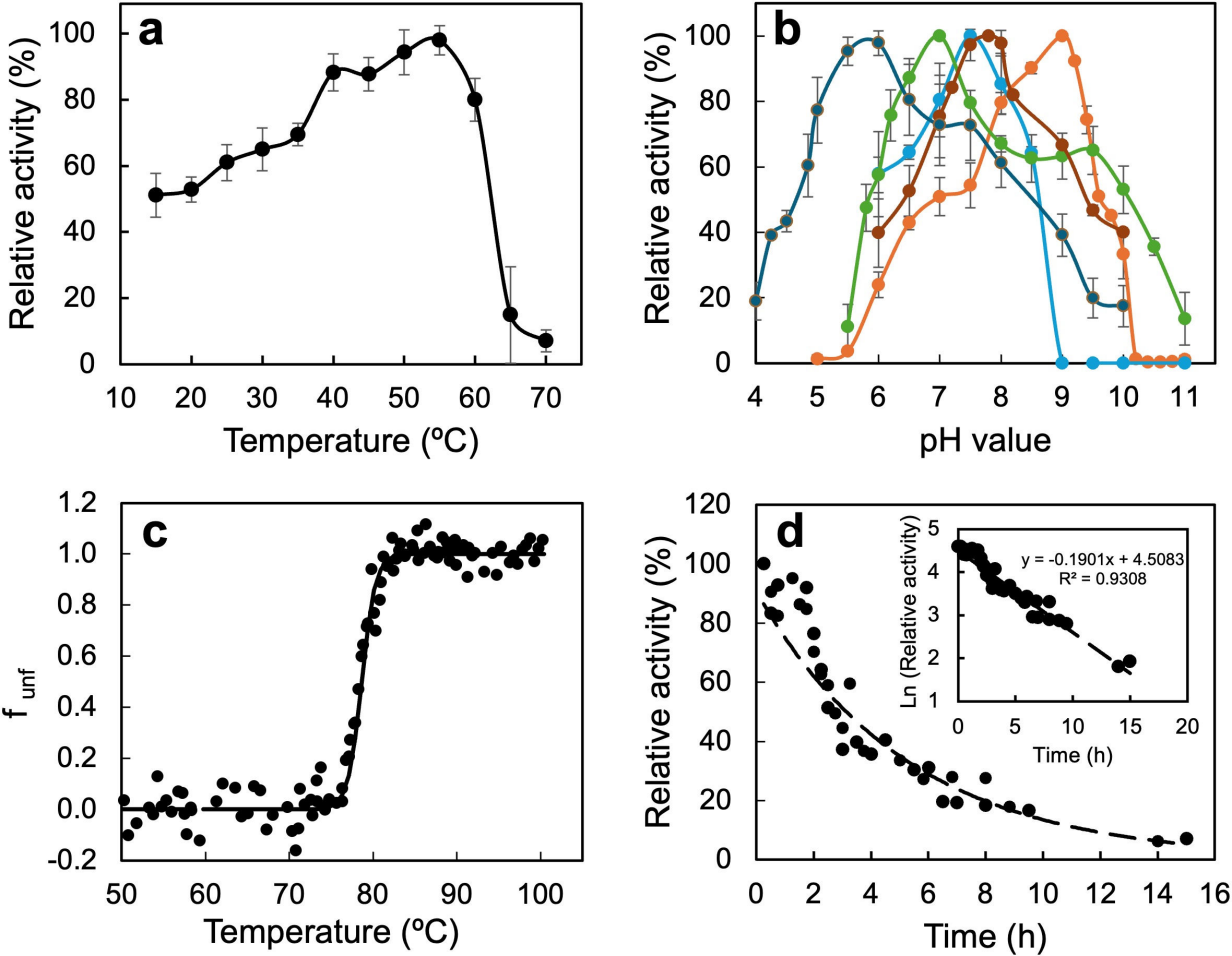
Temperature profile, pH profile, thermodynamic stability, and kinetic stability of PdG-D-LDH. (**a**) Temperature-dependent activity profile of PdG-D-LDH, measured between 15 and 80°C in 100 mM sodium phosphate buffer (pH 7.5) using 0.075 mM DCIP as the electron acceptor and 0.015 mg/mL of functional enzyme. (**b**) pH-dependent activity profile of PdG-D-LDH, assessed using different electron acceptors: 0.075 mM DCIP with 100 mM sodium L-lactate (orange), 0.4 mM 1,4-BQ with 100 mM sodium L-lactate (light blue), 0.5 mM NQS with 500 mM sodium L-lactate (dark blue), molecular oxygen via the Amplex Red assay with 20 mM sodium L-lactate (green), and 1 mM cytochrome-c with 50 mM sodium L-lactate (maroon). Colorimetric limitations affected measurements below pH 5, and auto-reduction of 1,4-BQ was observed above pH 9. (**c**) The thermal unfolding of the enzyme was measured by fluorescence emission. The solid line represents the fit of experimental data through the equation f_U_ = exp(−ΔG°/RT)/[1 + exp(−ΔG°/RT)]. The melting temperature was around 79 ± 1°C. The enthalpy change (ΔH) and the entropy change (ΔS) for thermal unfolding were 193 ± 68 kcal·mol^−1^ and 0.6 ± 0.2 kcal·mol^−1^·°C^−1^, respectively. (**d**) Kinetic stability of the PdG-D-LDH at 60°C. The inset shows the linear regression of logarithm activity vs time. The half-life of PdG-D-LDH at 60°C was 3.7 ± 0.1 h.

The enzyme exhibits maximum activity at 50–55°C, retaining 60% activity at 25°C (Fig. 3a), a feature like other D-LDHs from bacterial origin (Table S4). The pH dependence varied according to the electron acceptor used (Fig. 3b). The optimal pH ranged from 6 to 9, depending on the electron acceptor used: pH 6 for cytochrome-c (cyto-c), and between pH 7 and 8 for quinones and molecular oxygen, consistent with previously reported findings (Tables S4 to S6). In addition to the previously tested electron acceptors, cyto-c was also evaluated, as it has previously been reported to accept electrons from D-LDHs in other microbial strains (41), supporting the hypothesis that D-lactate oxidation by D-LDHs may be functionally linked to the mitochondrial respiratory chain, most likely through the direct transfer of electrons to cyto- c. For the artificial electron acceptor DCIP (pH_opt_ 9), comparable optimal pH values were found for other characterized D-LDHs from bacterial and archaeal origins (24, 42–44) (Table S4).

### Thermodynamic stability

The thermodynamic stability of the PdG-D-LDH was investigated by probing the tertiary structure using fluorescence spectroscopy at increasing temperatures and in the presence of increasing concentrations of the chemical denaturant guanidine hydrochloride (GdnHCl). The melting temperature (T_m_) corresponds to the point at which 50% of the protein population is in a folded state. It reflects the equilibrium between the native, functional protein and the unfolded state, providing a measure of thermodynamic stability. The PdG-D-LDH showed high thermostability with a T_m_ of 79 ± 1°C (Fig. 3c), outcompeting other characterized NAD-dependent D-LDHs (Table S3). The chemical unfolding profile was assessed to determine the mid-point of guanidine hydrochloride (GdnHCl) concentration at which 50% of the protein molecules were unfolded. The enzyme showed a mid-point of around 1.2 ± 0.2 M GdnHCl (Fig. S5), comparable to other microbial enzymes such as D-LDH from *Halobacterium marismortui* (45) and L-LDH from *Thermotoga maritima* (46). The kinetic or long-term stability is associated with the irreversible inactivation of the enzyme, and the half-life (t_1/2_) indicates the time required for the enzyme to lose 50% of its activity at a specific temperature. Enhancing kinetic stability is crucial for many biotechnological applications, as proteins often operate under challenging conditions, such as harsh extracellular environments or crowded intracellular settings, where harmful alterations can lead to enzyme inactivation (47). The half-life time was approximately 3.7 ± 0.1 h at 60°C as measured by thermal inactivation assays (Fig. 3d). This is a relatively high value, indicating that enzymes undergo irreversible denaturation at a relatively low rate, allowing them to remain functional for extended periods.

### Apparent steady-state kinetic characterization

The PdG-D-LDH activity was measured using L-lactate and D-lactate as electron donors and five electron acceptors: DCIP, 1,4-BQ, NQS, cyto-c, and oxygen (Table 2). The enzyme catalytic efficiency (*k_cat_*/K_m_) for D-lactate was two to three orders of magnitude higher than for L-lactate, regardless of the electron acceptor used. This finding confirms the stereoselectivity of the PdG-D-LDH, exhibiting a strong preference for D-lactate over L-lactate. This preference was primarily due to the enzyme’s higher affinity for D-lactate, which showed a significantly lower K_m_ for this substrate, ranging from 60 to 200 times lower than for L-lactate. The enzyme turnover number (*k_cat_*) values are comparable for all electron acceptors (either using L or D-lactate) (Table 2). Still, the highest activity was measured for DCIP at aerobic and anaerobic conditions. Among the closest related archaeal D-LDHs (see Fig. 1), none have been reported to exhibit activity for both enantiomers. This has only been observed in D-LDHs from the bacteria *Pseudomonas putida* KT2440 (48), *Corynebacterium glutamicum* (49), and *Paracoccus denitrificans* (50). This dual resemblance suggests that PdG-D-LDH may represent a unique evolutionary bridge between archaeal and bacterial LDHs.

**TABLE 2:** Apparent steady-state catalytic parameters of purified PdG-D-LDH for the oxidation of substrates sodium D- and L-lactate (electron donors)^*a*^

Electron donor	Electron acceptor	k_cat_^app^ (s^−1^)	K_m_ (mM)	*k_cat_ /* K_m_ (M^−1^·s^−1^)
D-lactate	DCIP	8.2 ± 0.3	1.0 ± 0.1	(8 ± 1) × 10^3^
	1,4-BQ	1.4 ± 0.1	0.52 ± 0.06	(2.6 ± 0.4) × 10^3^
	NQS	0.60 ± 0.02	0.71 ± 0.01	(1.1 ± 0.2) × 10^3^
	Cyto-c	0.029 ± 0.004	0.013 ± 0.004	(2.2 ± 1.0) × 10^3^
	Molecular oxygen	0.03 ± 0.01	0.016 ± 0.004	(1.9 ± 0.8) × 10^3^
L-lactate	DCIP	4.7 ± 0.2	59 ± 5	79 ± 6
	1,4-BQ	0.9 ± 0.2	147 ± 14	6.3 ± 1.4
	NQS	0.1 ± 0.0	53 ± 8	13 ± 2
	Cyto-c	0.03 ± 0.01	1.6 ± 0.1	19 ± 5
	Molecular oxygen	0.034 ± 0.001	9.5 ± 0.3	3.6 ± 0.3

^*a*^ Using DCIP, 1,4-BQ, NQS, cyto-c, and molecular oxygen as co-substrate (electron acceptors). The reactions were performed using DCIP and 100 mM glycine-NaOH at pH 9. For 1,4-BQ, NQS, and cyto-c, the reactions were conducted with 100 mM sodium phosphate buffer at pH 7.5. The response for oxygen (i.e., H_2_O_2_ formation) was performed using the Amplex Red assay with sodium phosphate buffer at pH 7.

Apparent steady-state kinetics were measured for 1,4-BQ, NQS, cyto-c, and oxygen, using constant concentrations of both L-lactate and D-lactate (Table 3). The enzyme exhibits four orders of magnitude higher catalytic efficiency for quinones than for cyto-c and molecular oxygen; 1,4-BQ is the best electron acceptor, regardless of whether the substrate is D- or L-lactate. The enzyme activity increased linearly with higher concentrations of both cyto-c and O_2_ (data not shown). In these conditions, even though *k*_cat_ and K_m_ cannot be determined separately, we can accurately extract the catalytic efficiency (k_cat_/K_m_). The catalytic efficiency for oxygen was approximately 800-fold lower than that for 1,4-BQ. The residual catalytic efficiency toward cyto-c is comparable to that observed for the D-LDH from *A. fulgidus*, a close phylogenetic relative of PdG-D-LDH. However, the latter may transfer electrons to native cytochromes (10). Comparing PdG-D-LDH kinetic parameters (Tables 2 and 3) with those of other D-LDHs is challenging due to variations in experimental conditions, such as temperature fluctuations and electron acceptor concentrations. Nevertheless, the values obtained are generally within the same range as those reported for enzymes from other sources (Tables S7 to S9).

**TABLE 3:** Apparent steady-state catalytic parameters of purified PdG-D-LDH for 1,4-BQ, NQS, cyto-c, and oxygen, using sodium D-lactate and L-lactate as substrates^*b*^

Electron acceptor	Electron donor	k_cat_^app^ (s^−1^)	K_m_ (mM)	*k_cat_ /* K_m_ (M^−1^·s^−1^)
1,4-BQ	D-lactate	1.8 ± 0.1	0.02 ± 0.01	(8 ± 3) x 10^4^
	L-lactate	1.3 ± 0.2	0.10 ± 0.05	(1.4 ± 0.6) x 10^4^
NQS	D-lactate	0.95 ± 0.04	0.08 ± 0.01	(1.2 ± 0.2) x 10^4^
	L-lactate	0.45 ± 0.03	0.3 ± 0.008	(1.8 ± 0.02) x 10^4^
Cyto-c	D-lactate	–^*c*^	–	12 ± 2^*a*^
	L-lactate	–	–	10 ± 1^*a*^
Molecular oxygen	D-lactate	–	–	100 ± 0^*a*^
	L-lactate	–	–	80 ± 14^*a*^

^*a*^ The *k_ca_*_t_/K_m_ was obtained from the first-order approximation of the Michaelis–Menten equation ([S] <<K_m_).

^*b*^ The reactions were performed using 1,4-BQ, NQS, and cyto-c with sodium phosphate buffer at pH 7.5 and oxygen in an oxygraph system apparatus.

^*c*^ –, not determined.

### Structural characterization

**Fig 4:**
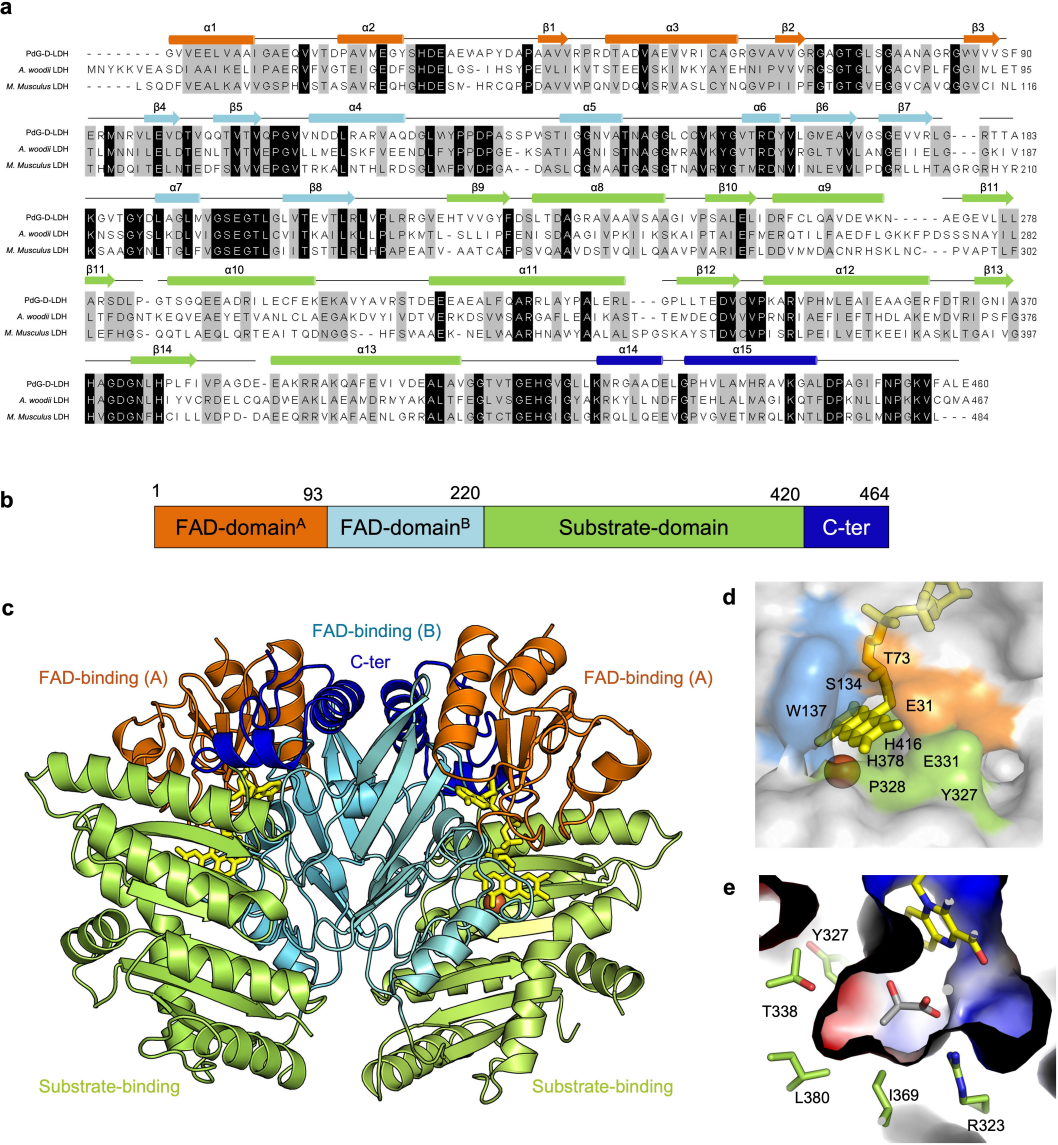
Amino acid sequence alignment of D-lactate dehydrogenases and active-site architecture of PdG-D-LDH. (**a**) Alignment based on 3D structural superpositions of PdG-D-LDH, *A. woodii* (PDB 7QH2, chain C), and *M. musculus* (PDB 8JDE) LDHs. Strictly conserved residues are marked in black boxes, whereas dark gray boxes represent the mostly conserved residues among the selected sequences. The different structural domains, namely α-helices and β-chains, are shown above the alignment. These are colored according to the domains they belong to: FAD-binding subdomains A (orange) and B (light blue), the substrate-binding domain (green), and the C-terminal domain (dark blue). (**b**) Schematic representation of the structural domains colored as in (**a**). (**c**) Cartoon representation of the PdG-D-LDH dimer colored as in (**a**). The FAD is shown as yellow sticks. The iron ion is shown as an orange sphere. (**d**) Zoomed view of the solvent-accessible surface of the FAD cavity. The residues lining the cavity are color-coded according to their respective domains. (**e**) The electrostatic surface of the substrate binding site was calculated by APBS Electrostatics Plugin, where red indicates negatively charged regions and blue indicates positively charged areas.

The crystal structure of the enzyme was solved both in the absence or presence of D-lactate (PdG-D-LDH–lactate), revealing a Cα root-mean-square deviation (RMSD) of 0.46 Å between the two structures (Fig. S6). The structure contains a non-covalently bound FAD at the active site. Moreover, a metal ion and the substrate D-lactate (in PdG-D-LDH-lactate complex) are clearly defined in the electron density (Fig. S7). PdG-D-LDH exhibits the characteristic VAO/PCMH structural fold (12, 30, 31). It is closer to *M. musculus* D-LDH (PDB 8JDE) with an RMSD of 1.57 Å than to *A. woodii* D-LDH (PDB 7QH2, chain C) with 1.91 Å (Fig. 4a), as previously mentioned when comparing the AlphaFold3 models (Table S1). PdG-D-LDH exhibits a higher RMSD (2.36 Å) relative to the third available X-ray crystal structure in the PDB database: the *E. coli* D-LDH structure (PDB 1F0X), likely reflecting the additional membrane-binding domain involved in transmembrane transport of amino acids and sugars (8).

The PdG-D-LDH structure contains a FAD-binding domain, a substrate-binding domain, and a C-terminal domain (Fig. 4a through c). The FAD-binding domain comprises subdomain A (residues 1–93), which contains a three-stranded parallel β-sheet (β1–β3) surrounded by three α-helices (α1–α3), and subdomain B (residues 94–216), which includes a five-stranded antiparallel β-sheet (β4–β8) surrounded by four α-helices (α4 and α7) (Fig. S8; Fig. 4a through c). The FAD-binding domain A contains a three-stranded parallel β-sheet (β1–β3) surrounded by three α-helices (α1–α3), while the FAD-binding domain B comprises a five-stranded antiparallel β-sheet (β4β8) surrounded by four α-helices (α4–α7). The substrate-binding domain consists of a six-stranded antiparallel β-sheet (β9–β14) enclosed by six α-helices (α8–α13). The C-terminal domain comprises two α-helices (α14–α15). The FAD is shown as sticks in yellow. The B-factor values of the substrate-binding domains are higher (36–126 Å^2^) than those of the other domains (25–85 Å^2^), indicating greater flexibility, which may play a role in binding substrates, product release, and catalysis (Fig. S9).

### Active site of PdG-D-LDH

The active site located at the interface of the FAD- and substrate-binding domains is connected to the solvent by a cavity with a volume of 314.6 Å^3^ and an area of 490.5 Å^2^ (Fig. 4d). Four out of ten residues present (E31, T73, H378, and H416) are strictly conserved in *M. musculus* and *A. woodii* D-LDHs (Fig. 4a). The substrate-binding site comprises two distinct regions: one predominantly positively charged, defined by R323, H371, H378, and H416, is located near the C1-carboxyl and C2-hydroxyl groups of D-lactate. The other, more hydrophobic, is primarily delimited by Y327, I369, and L380, facilitating a stronger interaction with the substrate (Fig. 4e). Most residues in this region are conserved in both PdG-D-LDH and m-D-LDH (Fig. 4a; Fig. S10).

**Fig 5:**
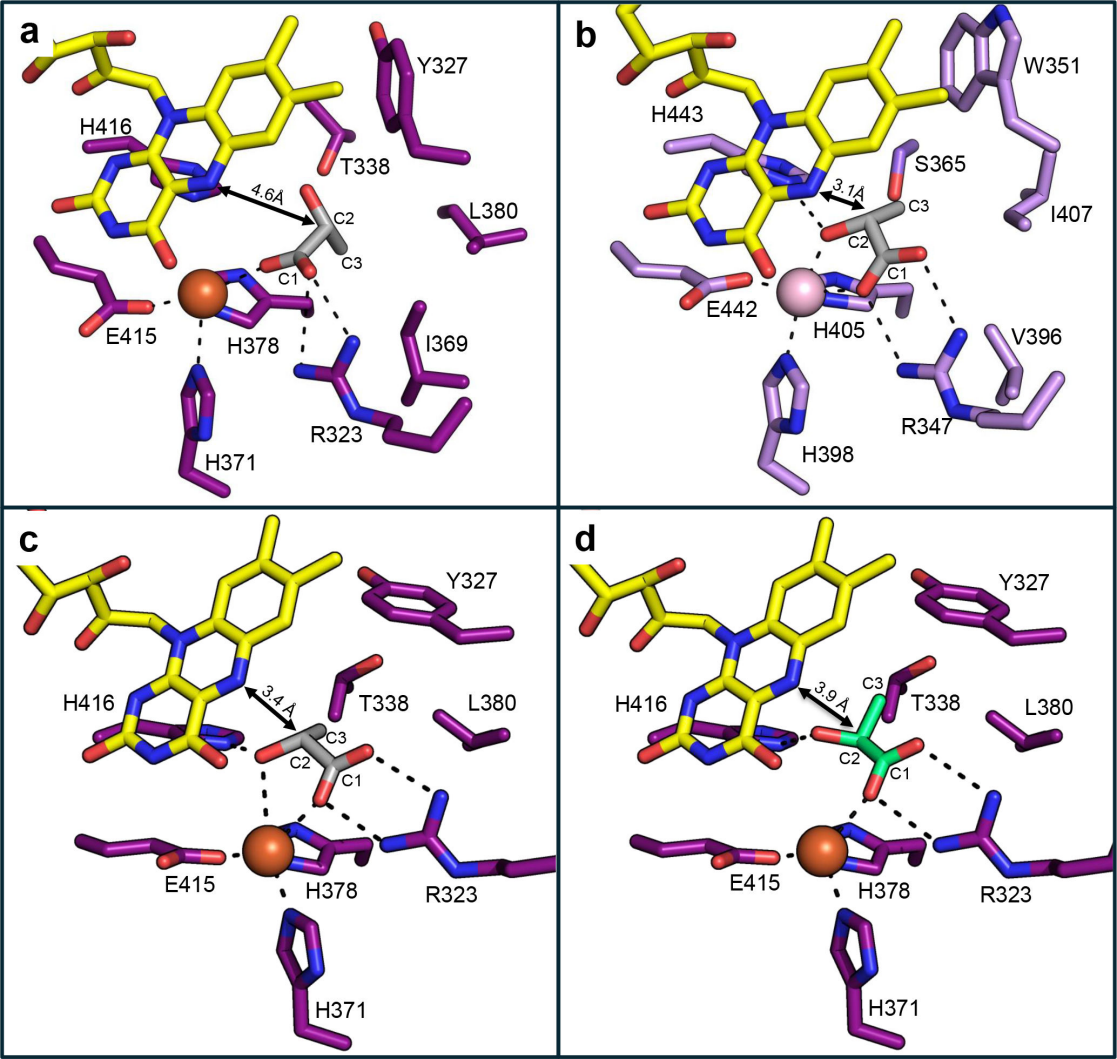
Substrate interactions on the active site. (**a**) Representation of the interactions between D-lactate and PdG-D-LDH residues. The distance (Å) between the C2 atom and the FAD^N5^ atom is marked with a black arrow. (**b**) Representation of the interactions between the D-lactate and residues in m-D-LDH (PDB 8JDE). Substrate docking of D-lactate (**c**) and L-lactate (**d**) in PdG-D-LDH. D-lactate and L-lactate carbon atoms are shown in gray and lime green, respectively. FAD carbon atoms are colored yellow, nitrogen atoms are blue, and oxygen atoms are red. The carbon atoms of the residues are shown in dark purple (PdG-D-LDH) and light purple (m-D-LDH). The iron and manganese ions are shown as orange and light pink spheres, respectively. The black dashed lines represent hydrogen bonds, salt bridges, and metal-ligand interactions.

The substrate-binding domain binds an Fe(II) ion coordinated by strictly conserved residues: two histidines (H371, H378) and one glutamate (E415) (Fig. 5a). The iron ion forms a hydrogen bond with the FAD O4 atom. In m-D-LDH (PDB 8JDE), a manganese ion is in the active site. After EDTA treatment followed by reconstitution with different divalent metal ions, the enzyme displayed the highest catalytic activity in the presence of Mn²^+^. In D-LDH, D-lactate binds Fe(II) and interacts with H416, which likely abstracts a proton from the C2-OH, enabling hydride transfer to FAD’s N5 (Fig. 5a). The C1 group coordinates iron and forms salt bridges with R323, while the C3 group engages in hydrophobic interactions with Y327, I369, and L380. In PdG-D-LDH, the C2-OH is 4.6 Å from FAD’s N5—too distant for efficient catalysis—unlike m-D-LDH, where this distance is 3.1 Å (Fig. 5b). Thus, the PdG-D-LDH–substrate complex likely lacks a catalytically competent geometry. Future studies will investigate whether the presence of Fe(II) in PdG-D-LDH reflects adaptation to the enzyme’s native environment and specific catalytic requirements, or whether its replacement with other metals (e.g., Mn(II)) could support substrate binding, modulate redox properties, and enhance the enzyme’s activity or stability under physiological conditions. Computational docking studies were performed in PdG-D-LDH to investigate the structural determinants underlying the distinct activity between L-lactate and D-lactate (Fig. 5c and d). In both substrates, the carboxylate group is stabilized by electrostatic interaction with R323, and a potential hydrophobic contact is observed between T338^CG2^ and the C3 group (3.8 Å and 4.0 Å, respectively). The CG2–C3 methyl groups in the D-lactate complex are better aligned for hydrophobic contact (Fig. 5c), suggesting a stronger interaction than in the L-isomer, consistent with its lower K_m_. Docking simulations suggest that T338 may adopt an alternative rotamer upon substrate binding, positioning its CG2 methyl group close to the C3 methyl group of D-lactate.

m-D-LDH exhibits a lower K_m_ and higher *k*_cat_/K_m_ for other organic acids with bulkier hydrophobic moieties (e.g., D-2-hydroxyvalerate and D-2-hydroxyhexanoate) compared to D-lactate, and shows no activity toward L-lactate (23). Docking studies revealed that in m-D-LDH, the C1 carboxyl and C2 hydroxyl groups are misaligned for optimal interactions with Mn(II) and surrounding residues. At the same time, its C3 group is poorly positioned for hydrophobic contacts, reducing binding and likely explaining the lack of activity (23). In contrast, the threonine found in PdG-D-LDH is replaced by serine (S365) in m-D-LDH (Fig. 5b). Although D-lactate adopts a catalytically competent pose in m-D-LDH, this appears to result from the overall hydrophobic environment of the pocket rather than a specific interaction with its methyl group.

### Bioproduction of pyruvate

**Fig 6:**
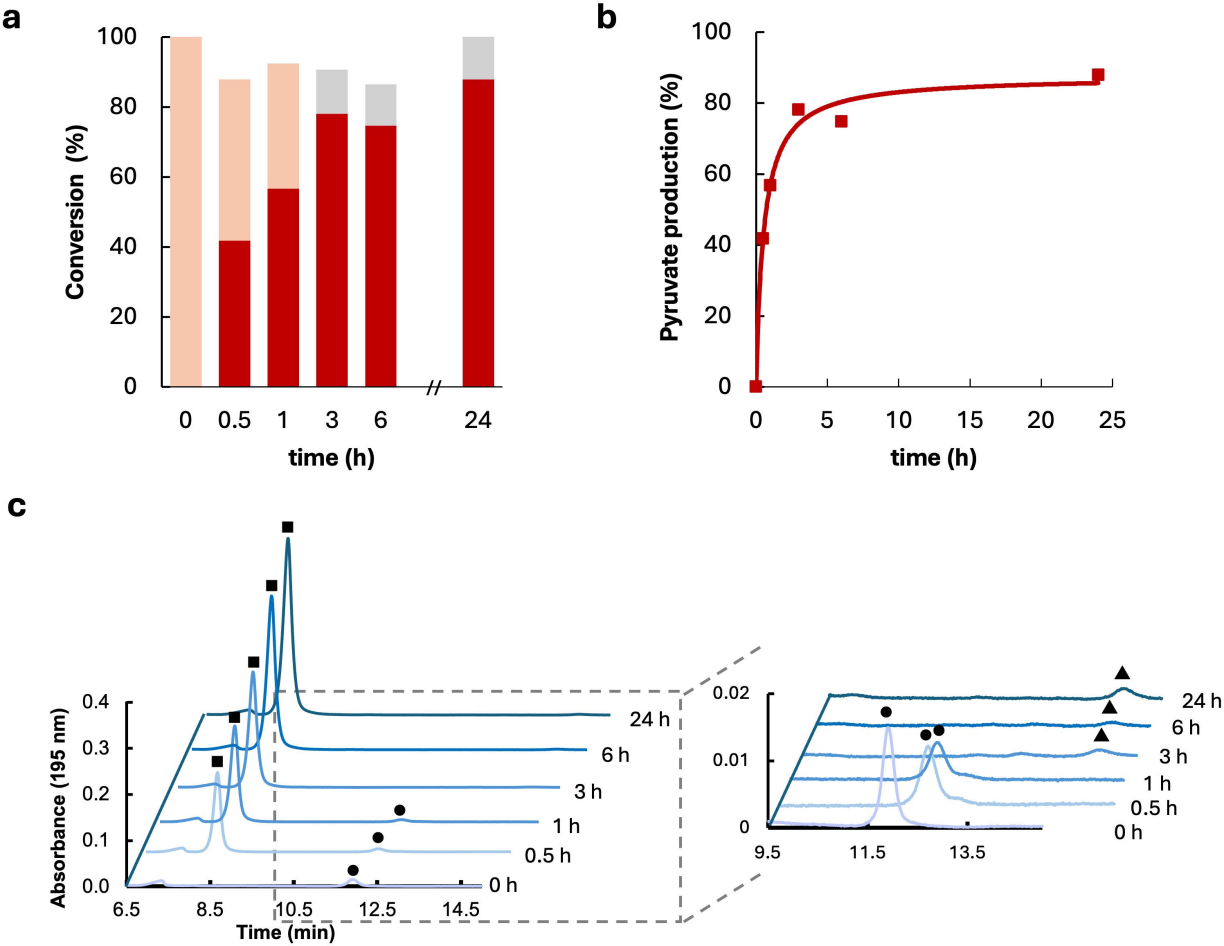
Bioconversion of D-lactate to pyruvate. (**a**) The bioconversion of 10 mM D-lactate to pyruvate in the presence of oxygen as an electron acceptor with 5 U/mL enzyme at 35°C in the presence of 300 U/ml catalase. After 24 h, the 10 mM of D-lactate (pink) were converted into pyruvate (red) and acetate (gray). (**b**) Pyruvate production versus time of reaction. (**c**) The HPLC chromatograms of the reaction in the presence of catalase. After 3 h, 10 mM of D-lactate (circle) was converted into 9 mM pyruvate (square) and 1 mM of acetate (triangle).

Reaction conditions were optimized to evaluate the potential of PdG-D-LDH as a biocatalyst in the bioconversion of lactate to pyruvate. This central metabolic intermediate can be converted into a range of valuable compounds, making it a practical focus for many biotechnological applications. We first tested the enzyme using D-lactate and 1,4-butanediol quinone (1,4-BQ) as substrates. However, after 24 h of reaction, 90% of the product was identified as acetate, and only 10% was pyruvate (Fig. S11a). These data suggest that under these conditions, the enzyme functions as a lactate oxidase, i.e., it uses oxygen as an electron acceptor, producing pyruvate and hydrogen peroxide, which then react non-enzymatically, leading to the formation of acetate, CO₂, and water, as previously described (12). This result was confirmed in reactions without 1,4-BQ, where a nearly identical product distribution was observed (Fig. S11b). We found that the enzyme is inhibited by concentrations of 1,4-BQ above 1 mM, which can explain its low LDH activity under the reaction conditions (10 mM pyruvate at aerobic conditions) and the use of molecular oxygen as an electron acceptor instead (Fig. S12). Therefore, reactions were carried out using oxygen as the electron acceptor, resulting in a substantial increase in pyruvate yield—from 10% to 90% after 24 h (Fig. 6). The addition of catalase was necessary to degrade the hydrogen peroxide formed during the reaction and to prevent acetate formation (data not shown). The high pyruvate yields achieved with PdG-D-LDH are notable compared to previously reported values in the literature, where yields from D-lactate reached only 19.5% and 50% (43, 51). Remarkably, the enzyme exhibits an IC50 for pyruvate of 53 mM (Fig. S13), surpassing other enzymes investigated, particularly LOX from *Enterococcus hermanniensis*, which loses 50% of its activity at just 0.7 mM pyruvate (17, 18, 52). PdG-D-LDH catalyzes the oxidation of D-lactate to pyruvate using oxygen as the electron acceptor, unlike previous studies that relied on artificial acceptors (17). Despite oxygen’s low solubility, the enzyme’s low K_m_ enables efficient conversion under mild conditions. While substrate cost and enzyme loading remain limitations, racemic DL-lactate can be used directly, and enzyme engineering may further improve process feasibility. Furthermore, recent advances in microbial production of D-lactate from lignocellulosic wastes (13, 53) are expected to substantially reduce D-lactate costs, further improving the overall economic feasibility of selective processes. This study provides a proof of concept for the selective formation of pyruvate and a basis for future biocatalysis studies.

### Conclusions

Shotgun metagenomic sequencing of *P. dilatatus* revealed an abundance of pathways related to carbohydrate and energy metabolism. A new FAD-dependent D-lactate dehydrogenase (PdG-D-LDH) was successfully identified, expressed, produced, purified, and characterized. This enzyme preferentially oxidizes D-lactate over L-lactate with a catalytic efficiency difference of three orders of magnitude, exhibits thermophilic behavior (optimum at 55°C, melting temperature ~80°C), and shows high thermal stability (half-life ~4 h at 60°C), making it well suited for industrial applications. X-ray crystal studies and *in silico* analysis show that the enzyme’s active site features a solvent-accessible cavity where the FAD cofactor, Fe(II), and key residues collaborate to stabilize the substrate and facilitate catalysis. Favorable hydrophobic interactions and optimal alignment facilitate hydride transfer, accounting for its high catalytic efficiency in the reduction of D-lactate. Given its ability to convert D-lactate into pyruvate under mild conditions, PdG-D-LDH provides a proof of concept for lactate oxidation by biocatalysis and expands the repertoire of known D-LDHs. These findings support the continued exploration of microbial enzymes from underexplored niches and demonstrate how metagenomics, structural biology, and computational tools can accelerate the discovery of biocatalysts for sustainable biotechnological applications.

## MATERIALS AND METHODS

### *P. dilatatus* collection and dissection

A representative number of *P. dilatatus* individuals (~15 individuals) were collected from two cellulosic biomass-rich environments in Coimbra, Portugal: the National Forest of Choupal (40°13′19″N 8°26′46″W) and the Botanical Garden of the University of Coimbra (40°12 22.493"N 8° 25' 26.778" W). The isopods were stored in plastic boxes and kept on ice to preserve their gut content during transport. The isopods were washed with sterile distilled water to remove any contaminating surface soil debris and anesthetized with a cotton ball soaked in chloroform for 30 min (54). Under a magnifying glass, the heads, legs, and hepatopancreas were removed with tweezers and a scalpel. Then, the gut (from the esophagus to the rectum) was carefully separated from the rest of the digestive tract and cut longitudinally with a scalpel. Each gut was cut longitudinally and placed separately in 1.5 mL tubes containing 500 µL of phosphate-buffered saline (PBS) solution (pH 7.2). To separate gut content from epithelial cell debris, the samples were vigorously vortexed, and the gut epithelial cells were removed (55). The resultant gut content suspension was used to extract total DNA.

### Extraction of genomic DNA

The genomic DNA from each *P. dilatatus* gut sample was extracted using the PowerSoil DNA Isolation Kit (MO BIO Laboratories Inc., CA, USA) according to the manufacturer’s instructions. The genomic DNA concentration and quality were determined using a Nanodrop spectrophotometer (Thermo Scientific) and assessed by 1% agarose gel electrophoresis, respectively.

### Next-generation sequencing: 16S rRNA sequencing

According to the manufacturer’s protocols, the genomic DNA of eleven *P. dilatatus* gut samples was sequenced using the bacterial 16S rRNA gene amplicon sequencing method on an Illumina MiSeq V2 platform. The 16S rRNA sequencing was done by Eurofins (Germany). The structural diversity of the communities was determined by amplifying the hypervariable regions V1–V3 of the bacterial 16S rRNA gene using the primers 28F-GAGTTTGATCNTGGCTCAG-, 519R–519R-GTNTTACNGCGGCKGCTG-. Raw data were analyzed using the mothur v.1.47 software package (http://www.mothur.org) (accessed on 20 January 2023) (56). Briefly, sequences were subjected to conservative quality control measures during the initial quality trimming and assembly of contig read sequences, using an *in-house* script. The resultant sequences were aligned and clustered into operational taxonomic units (OTUs) with a cutoff of 0.03 (97% similarity). All high-quality sequences were taxonomically classified with the ARB-Silva taxonomic database 138 (57, 58). This analysis identified and excluded gut samples dominated by specific bacterial species, namely *Wolbachia*, *Candidatus Hepatoplasma*, and *Rickettsiella* (19). Excluding the most abundant samples helps ensure a focus on the core gut microbiota and maintains the essential bacterial communities that contribute most to the organism’s health and function.

### Next-generation sequencing shotgun metagenome analysis

After a brief analysis of the structural diversity of microbial communities, the genomic DNA from six *P. dilatatus* gut samples was pooled and subdivided into three aliquots, which were sequenced in parallel. Shotgun metagenomic sequencing was performed at Eurofins (Germany). The obtained paired-end sequences were assembled using metaSPAdes (59). The Prodigal v2.6.3 software (60), operating in metagenome mode, was used to identify the open reading frames (ORFs). The taxonomic and functional profile of *P. dilatatus* gut metagenome was determined using the Ghostkoala software against the KEGG database (61).

### Genome mining and identification of D-lactate dehydrogenase encoding genes

Two D-lactate dehydrogenases were used as query sequences because they have available biochemical and structural data (PDB 8JDE [23] and PDB 7QH2, chain C [25]). A local protein-protein BLAST search was performed using the Blast standalone tool against the “PdG.Database.” An *in-house* script was used to process the results, identifying five sequences with the highest similarity to the query sequences. These sequences included the nearest complete matches without non-proteinogenic residues (“X”) (Table S1). The final selection was based on the sequence that most reliably corresponded to a D-lactate dehydrogenase, as it appeared in the top five predictions for both query sequences.

### *In silico* analysis of the sequence candidates

The selected sequence, PdG-D-LDH, was subjected to *in silico* analysis to predict its structural, functional, and biochemical characteristics. The predicted structural model of PdG-D-LDH was generated using the AlphaFold3 server (21). The best model was ranked according to its predicted local-distance difference test (pLDDT) confidence values. The machine learning tool CLEAN (62) was used to indicate the function of the candidate sequence. The Protparam tool (Expasy) (63) was used to estimate biochemical parameters, including molecular weight, instability index, and theoretical isoelectric point. The melting temperature (T_m_) was predicted using the T_m_ predictor program (64).

### Phylogenetic analysis

Amino acid sequences of several lactate oxidases (LOX) and dehydrogenases (LDH) experimentally characterized and reported in the literature (Table S2) were used to explore evolutionary relationships. The eugenol oxidase (EUGO) from *Rhodococcus jostii* (Q0SBK1) (35) was used as the outer group. All the sequences were downloaded from the NCBI database. The sequence similarity analysis was performed using the MEGA (Molecular Evolutionary Genetics Analysis) software version 11. The phylogenetic tree was constructed using the maximum likelihood method with 500 bootstrap replicates (65). The phylogenetic tree was visualized and annotated using iTOL (Interactive Tree of Life) (36).

### Bacterial strains, plasmids, and culture media

The PdG-D-LDH gene, cloned into the pET15 b vector, was synthesized by Genescript (NJ, USA) with a hexahistidine tag at the N-terminus and codons optimized for use in *E. coli*. The *E. coli* DH5α strain (Novagen) was used for routine propagation and amplification of plasmid constructs, and the *E. coli* Tuner strain was used for heterologous expression. Luria-Bertani (LB) was used as a routine liquid culture medium, and Luria-Bertani agar (LA) was used as a solid medium. All media were supplemented with 100 µg⋅mL^−1^ of ampicillin for cell culture.

### Production and purification of PdG-D-LDH

The recombinant strain of *E. coli* Tuner carrying the plasmid pET15-b with the PdG-D-LDH gene was grown in 2.5 L of LB medium supplemented with 100 µg × mL^−1^ of ampicillin (NZYTech) in Corning 5 L Baffled PETG Erlenmeyer flasks. The initial OD_600nm_ was 0.05, and growth proceeded at 37°C and 110 rpm until it reached an OD_600nm_ of 0.7. The gene expression was induced by adding 0.1 mM IPTG (NZYTech) to the culture medium, and the temperature was lowered to 25°C, while the culture was stirred. The culture was then left overnight. The cells were harvested by centrifugation (4420 × *g*, 10 min, 4°C). The cells were resuspended in 20 mM Tris-HCl buffer (pH 7.6) containing 200 mM NaCl, 0.001 U/mL DNAse I, 5 mM MgCl_2_, and a mixture of protease inhibitors (1.75 mM antipain from Carl Roth and 2.50 mM leupeptin from Carl Roth). Cells were then disrupted using a French Press cell operating at 1,000 psi for three cycles. The cell debris was removed by centrifugation at 39,191 × *g* for 1 h at 4°C, and the supernatant was collected and filtered (0.22 µm pore). Purification was performed using a Histrap HP column (Cytiva, Life Science) equilibrated with 20 mM Tris-HCl buffer (pH 7.6) containing 200 mM NaCl and 40 mM imidazole (Sigma-Aldrich). The elution was performed using a one-step linear gradient of 500 mM imidazole over five column volumes at a flow rate of 1 mL·min^−1^. The eluted yellow fractions were concentrated and loaded onto a HiLoad Superdex 200 column (Cytiva, Life Sciences), pre-equilibrated with 20 mM Tris-HCl (pH 7.6) containing 200 mM NaCl, at a flow rate of 1 mL·min^−1^. The yellow fractions corresponding to the dimeric form were collected. For storage at −20°C, 20% glycerol was added. The total protein concentration was estimated using the Bradford assay with bovine serum albumin as a standard. The functional protein concentration was assessed by measuring the FAD content, using Lambert–Beer’s law from the absorbance measurements (at 450 nm) and the extinction coefficient of FAD (ε450 nm = 11,300 M^−1^·cm^−1^). The preparation purity was assessed through SDS-PAGE.

### Determination of oligomerization state

Size exclusion chromatography (SEC) using a Superdex 200 10/300 Gl column was employed to assess the oligomerization state of the enzyme in solution. The chromatography was conducted using a 20 mM Tris-HCl buffer (pH 7.6) containing 200 mM NaCl, at a flow rate of 1 mL/min. Molecular weight standards (Biorad) used for calibration included thyroglobulin (670 kDa), gamma globulin (158 kDa), ovalbumin (44 kDa), and myoglobin (17 kDa).

### Identification of enzyme cofactor

Ultraviolet-Visible (UV-vis) spectroscopy was used to detect the characteristic flavin absorption band and assess the type of cofactor attachment. The absorption spectrum of the purified enzyme (at 15 µM) was recorded before and after treatment with 0.4% SDS using a Shimadzu UV-1603 spectrophotometer, as previously described (66). To determine the specific flavin cofactor present, a 100 µM enzyme solution was denatured at 100°C for 10 min to release the bound flavin. Following denaturation, protein precipitates were removed by centrifugation (14,550 × *g*, 10 min, 4°C), and the supernatant was analyzed via high-performance liquid chromatography (HPLC) using a previously described method (67). The runnings were performed using a LichroCART 100 RP-18 column (125 × 4 mm, 5 µm particle size; Merck KGaA, Darmstadt, Germany) on a Waters Alliance 2695 HPLC System (Milford, MA, USA) coupled with a Waters Photodiode Array Detector 2996, operated by Empower Pro version 5 (2002) software (Waters Chromatography, Milford, MA, USA). The experiments were conducted under a two-solvent system consisting of 5 mM ammonium acetate buffer (pH 6.5) (as solvent A), and methanol (as solvent B). The running buffer consisted of 85% solvent A and 15% solvent B, operating at 40°C with a flow rate of 1 mL·min^−1^. Five minutes after the injection, elution was performed using a linear gradient from 15% to 75% B in 20 min. The column was then regenerated by applying 100% solvent B. The flavins were detected by absorption at 264 nm. Standards used were for flavin mononucleotide (FMN, Sigma-Aldrich) and flavin adenine dinucleotide (FAD, Acros Organics).

### Determination of iron content

ICP-OES (inductively coupled plasma atomic emission spectroscopy) estimated the amount of iron in protein preparations at the Central Analysis Lab of Universidade de Aveiro (Aveiro, Portugal) using an enzyme preparation at around 155 µM.

### Screening of electron acceptors using UV-Vis

An enzyme solution at around 12 µM in 20 mM Tris-HCl (pH 7.6) and 200 mM NaCl was reduced with 10 equivalents of dithionite. The reoxidation of the flavin was monitored via UV-vis spectroscopy after the addition of various electron acceptors: nicotinamide adenine dinucleotide (NAD, TCI chemicals, 1 mM), nicotinamide adenine dinucleotide phosphate (NADP, Sigma-Aldrich, 1 mM), 1,4-benzoquinone (1,4-BQ, Alfa Aesar, 1 mM), 1,2-naphthoquinone-4-sulfonic acid (NQS, Alfa Aesar, 2 mM), 2,6-dichlorophenolindophenol (DCIP, Sigma-Aldrich, 0.075 mM), and molecular oxygen (pure oxygen was bubbled directly into the solution). This procedure was not used with cytochrome c from equine heart (cyto-c, Sigma-Aldrich) because the reducing agent (dithionite, Sigma-Aldrich) would also reduce the cytochrome in the reaction mixture, thereby interfering with the assay. Enzyme spectra were recorded before and after the addition of each electron acceptor. The spectra were monitored for at least 1 h. All experiments were performed at room temperature, and anaerobic conditions were achieved by purging solutions and enzyme preparations with argon.

### Screening of electron donors using enzymatic assays

A screening of substrates was performed, including several acids such as L-lactate (Thermo Scientific), D-lactate (Sigma-Aldrich), maleic acid (Sigma-Aldrich), fumaric acid (Alfa Aesar), succinic acid (Merck), DL-malic acid (Sigma-Aldrich), L-tartaric acid (Merck), gallic acid (Merck), mandelic acid (Sigma-Aldrich), vanillin acid (Sigma-Aldrich), cinnamic acid (Sigma-Aldrich), *p*-coumaric acid (Sigma-Aldrich), *m*-coumaric acid (Sigma-Aldrich), *o*-coumaric acid (Sigma-Aldrich), caffeic acid (TCI chemicals), syringic acid (Sigma-Aldrich), ferulic acid (Sigma-Aldrich), sinapic acid (Alfa Aesar), palmitic acid (Sigma-Aldrich), and oleic acid (Sigma-Aldrich). Alcohols used included glycerol (Fisher), furfuryl alcohol (Sigma-Aldrich), resorcinol (Analar), benzyl alcohol (Thermo Scientific), pyrogallol (Sigma-Aldrich), guaiacol (Sigma-Aldrich), 4-aminobenzyl alcohol (Thermo Scientific), anisyl alcohol (Honeywell Fluka), vanillyl alcohol (Alfa Aesar), syringol (Alfa Aesar), 2-phenylethanol (Merck), veratryl alcohol (Sigma-Aldrich), pyrocatechol (Honeywell Fluka), eugenol (Sigma-Aldrich), coniferyl alcohol (Thermo Scientific), sinapyl alcohol (Sigma-Aldrich), and 3,4,5-trimethoxybenzyl alcohol (Sigma-Aldrich). Sugars included arabinose (PanReac Applichem), ribose (VWR), fucose (Carl Roth), mannose (Alfa Aesar), sorbose (MP Biomedicals), xylose (Sigma-Aldrich), galactose (Sigma-Aldrich), rhamnose (PanReac Applichem), glucose (PanReac Applichem), fructose (Sigma-Aldrich), glucosamine (Sigma-Aldrich), cellobiose (BDH), trehalose (Sigma-Aldrich), maltose (Sigma-Aldrich), melibiose (Alfa Aesar), palatinose (Sigma-Aldrich), raffinose (Alfa Aesar), and melezitose (Sigma-Aldrich). Aldehydes and furans include furfural aldehyde, hydroxymethylfurfural (HMF, Sigma-Aldrich), 5-formyl-2-furan carboxylic acid (FFCA, Sigma-Aldrich), and 2,5-diformylfuran (DFF, Sigma-Aldrich). The reaction mixtures contained 1 or 10 mM electron donor substrate and 0.075 mM DCIP in 100 mM sodium phosphate buffer (pH 7.5). All assays were performed in 96-well microplates in a final volume of 200 µL at 25°C. The reduction of DCIP was monitored by measuring the absorbance at 600 nm (ε_600 nm_ = 8.98 mM^−1^·cm^−1^) using a Synergy microplate reader. One unit (U) of enzyme activity was defined as the oxidation of 1 µmol of substrate per minute.

### Temperature profile

The temperature dependence of PdG-D-LDH activity was evaluated between 15°C and 70°C, with 5°C intervals. The reaction mixture consisted of 100 mM sodium L-lactate, 0.075 mM DCIP, and 100 mM sodium phosphate buffer (pH 7.5), and was initiated by adding 0.015 mg⋅mL^−1^ of the functional enzyme. Reactions were monitored at 600 nm using a Cary 100 Bio UV-Vis Multicell spectrophotometer.

### pH profile

The pH dependence of enzyme activity was evaluated using five electron acceptors: DCIP, 1,4-BQ, NQS, cyto-c, and molecular oxygen (measured via the Amplex Red-HRP assay). Activity was assessed across a pH range of 4.5 to 12 using Britton-Robinson buffer. For the DCIP assay, the reactions contained 100 mM sodium L-lactate and 0.075 mM DCIP and were monitored at 600 nm. For 1,4-BQ and NQS, reactions included 500 mM sodium L-lactate with either 0.4 mM 1,4-BQ or 0.5 mM NQS. They were monitored at 290 nm (ε_290nm_ = 9.3 mM⁻¹·cm⁻¹ for hydroquinone, the product of 1,4-BQ reduction) or at 2.6 mM⁻¹·cm⁻¹ (for dihydro-naphthoquinone, the product of NQS reduction). For the cyto-c assay, reactions contained 50 mM sodium L-lactate and 1 mM cyto-c and were monitored at 550 nm (ε_550nm_ = 28 mM⁻¹·cm⁻¹, corresponding to the reduced cyto-c). Oxygen consumption was measured using the Amplex Red-HRP assay, which produces the fluorescent product resorufin (ε_560nm_ = 54 mM⁻¹·cm⁻¹). The reactions for oxygen contained 20 mM sodium L-lactate, 8 U·mL⁻¹ horseradish peroxidase, and 0.05 mM Amplex Red (10-acetyl-3,7-dihydroxyphenoxazine, Chemodex), and absorbance at 560 nm was monitored to track resorufin formation.

### Apparent steady-state kinetics for L- and D-lactate

The catalytic parameters of PdG-D-LDH were assessed using two electron donors (L- and D-lactate) and five electron acceptors: DCIP, 1,4-BQ, NQS, cyto-c, and molecular oxygen. Saturating concentrations of electron acceptors were used for 1,4-BQ and NQS. Limitations in the spectrophotometric method prevented the accurate determination of DCIP above 0.3 mM and cyto-c above 1 mM. For oxygen, the constant concentration was ~ 250 µM. When DCIP was used, the reaction contained 0.075 mM DCIP and either 0–200 mM L-lactate or 0–10 mM D-lactate in 100 mM glycine–NaOH buffer (pH 9). The reaction was monitored at an absorbance of 600 nm. When 1,4-BQ was used as the electron acceptor, reactions contained 0.4 mM 1,4-BQ and 0–750 mM L-lactate or 0.1 mM 1,4-BQ with 0–100 mM D-lactate. The reactions were performed in a 100 mM phosphate buffer (pH 7.5) and monitored at 290 nm. Reactions using NQS were performed with 0.5 mM NQS in the presence of 0–750 mM sodium L-lactate or 0–20 mM sodium D-lactate in 100 mM phosphate buffer (pH 7.5) and monitored at 290 nm. For the electron acceptor cyto-c, reactions contained 1 mM cyto-c and 0–50 mM L-lactate or 0–2 mM D-lactate. The reactions occurred in 100 mM phosphate buffer (pH 7.5) and were monitored at an absorbance of 550 nm. When the molecular oxygen was used as an electron acceptor, reactions containing 8 U·mL^−1^ HRP and 0.05 mM Amplex Red were monitored at 560 nm. All reactions were performed using 0.02–0.2 μM of pure enzyme at 25°C using a Synergy2 microplate reader. The catalytic parameters were determined by fitting the data to the Michaelis–Menten equation using Origin95 software.

### Apparent steady-state kinetics for different electron acceptors

A constant saturating concentration of L-lactate or D-lactate was used to determine each electron acceptor’s apparent steady-state kinetic parameters. For DCIP, catalytic parameters were estimated from reactions containing 0–0.3 mM DCIP and 200 mM sodium L-lactate or 10 mM sodium D-lactate in 100 mM glycine-NaOH buffer (pH 9). For 1,4-BQ, reactions were conducted with 0–0.4 mM 1,4-BQ and 500 mM L-lactate or 0-0.1 mM 1,4-BQ and 20 mM D-lactate in 100 mM phosphate buffer (pH 7.5). For NQS, the reaction mixture contains 0–0.5 mM NQS and 500 mM sodium L-lactate or 20 mM sodium D-lactate in 100 mM phosphate buffer (pH 7.5). For cyto-c, the mixtures contained 0–1 mM cyto-c and 50 mM sodium L-lactate or 10 mM sodium D-lactate in 100 mM phosphate buffer (pH 7.5). All reactions were conducted at 25°C and monitored spectrophotometrically at the specified wavelength using a Synergy2 microplate reader (BioTek, Vermont, USA). For molecular oxygen, enzymatic rates were measured using an oxygraph system (Hansatech Instruments, Pentney, UK) calibrated at 25°C with a buffer saturated with air. The reaction mixture contained 0.2 mM D-lactate (or 20 mM L-lactate) in 100 mM phosphate buffer (pH 7.5) under continuous stirring (40 rpm). The oxygen concentration was controlled by bubbling a mixture of O_2_ or N_2_ gases into the reaction cell. The apparent steady-state catalytic parameters were determined by fitting the data to the Michaelis–Menten equation using Origin95 software (OriginLab, Northampton, MA, USA) or the first-order approximation of the Michaelis-menten equation ([S]<<K_m_).

### Enzyme stability

The kinetic stability studies were performed as previously described (68). Briefly, the enzyme was incubated at 60°C in 20 mM Tris-HCl buffer containing 200 mM NaCl (pH 7.6). At appropriate time points, aliquots were withdrawn, cooled, and tested for activity using 10 mM D-lactate as the electron donor and 0.075 mM DCIP as the electron acceptor. The inactivation constants (k_in_) were obtained by linear regression of the logarithm of activity versus time. The half-life (t_1/2_) was calculated using the equation t_1/2_ = ln2/k_in_. The thermostability and chemical stability of the enzyme were assessed using steady-state fluorescence, measured with a Cary Eclipse spectrofluorometer (Agilent Technologies) at an excitation wavelength of 296 nm (47). For thermostability, the fluorescence data were recorded over a temperature range from 20°C to 100°C, with the temperature increasing at 1°C per minute, and emission was monitored at 340 nm. For chemical stability, guanidine hydrochloride (GdnHCl) at concentrations ranging from 0 to 4 M in 20 mM Tris-HCl buffer containing 200 mM NaCl (pH 7.6) was used to induce protein unfolding. The fluorescence emission was recorded between 310 and 450 nm, and all experiments were performed at room temperature. The chemical-induced unfolding was analyzed using a two-state process (47). All stability assays were performed, at least in triplicate, in 20 mM Tris-HCl buffer containing 200 mM NaCl (pH 7.6).

### Protein crystallization

Initial crystallization screenings of the PdG-D-LDH were performed using the sitting-drop vapor diffusion method at 293 K, employing 96-well sitting-drop iQ plates with a Mosquito LCP crystallization robot (TTP Labtech, Cambridge, UK). The BCS Screen (Molecular Dimensions) resulted in the formation of yellow, plate-like PdG-D-LDH crystals within 10 days, using 0.2 M sodium formate, 0.1 M sodium phosphate (pH 6.2), 20% (vol/vol) PEG Smear Medium, and 10% (vol/vol) glycerol. The condition was optimized using the hanging drop vapor diffusion method in XRL 24-well crystallization plates with 500 µL of the reservoir solution (Molecular Dimensions, Newmarket, UK), where the protein: precipitant ratio and precipitant concentration were screened. An additive screen (Hampton Research) was performed, and better diffracting crystals were obtained in condition G3 (30% [vol/vol] 2-methyl-2,4-pentanediol, MPD). The final optimized crystallization condition for PdG-D-LDH contained 2 µL of protein (15 mg/mL), 0.5 µL of 0.2 M sodium formate, 0.1 M sodium phosphate (pH 6.2), 20% (vol/vol) PEG Smear Medium and 10% (vol/vol) glycerol, and 0.5 µL of 30% (vol/vol) 2-methyl-2,4-pentanediol. Crystals of the PdG-D-LDH-substrate complex were obtained by soaking PdG-D-LDH crystals in the reservoir solution containing 20 mM D-lactate for 10 min. As the crystallization solution was fully cryoprotectant, the crystals were immediately flash-cooled in liquid nitrogen.

### X-ray diffraction data collection and processing

PdG-D-LDH and PdG-D-LDH-lactate complex diffraction data were measured at the ID30B beamline of the European Synchrotron Radiation Facility, ESRF (Grenoble, France) and the XALOC beamline BL13 of the ALBA Synchrotron (Barcelona, Spain), respectively (69). The diffraction data of PdG-D-LDH were obtained using an EIGER2 × 9M detector and 0.873128 radiation wavelength, crystal-to-detector distance of 403 mm, and oscillation width of 0.20° Å in a total of 180° rotation. PdG-D-LDH-lactate complex diffraction images were obtained with a PILATUS 6M detector, using 0.979180 Å radiation wavelength, crystal-to-detector distance 576 mm, and oscillation width 0.10° in a total of 166° rotation. Data were indexed and integrated with XDS (70). The space group was determined using POINTLESS (71), and the data were scaled using AIMLESS (71, 72). These programs were used within the autoPROC data processing pipeline (73). Data collection and processing statistics are listed in Table S10.

### Structure determination and refinement

Matthews coefficient calculations (74) indicated a high probability of eight molecules in the asymmetric unit for PdG-D-LDH and the PdG-D-LDH-lactate complex crystals. The phase problem of both structures was solved by molecular replacement using the PdG-D-LDH AlphaFold-predicted model as a search model and PHASER (75), which is included in the PHENIX suite (76). Automated model rebuilding and completion were performed with PHENIX.AUTOBUILD (76), followed by manual model building and iterative refinement cycles using PHENIX.REFINE (77–79) and COOT (80). Refinement included atomic positions, isotropic atomic displacement parameters (a.d.p.s), automatic solvent waters modeling, as well as polypeptide chain regions of translation, libration, and screw refinement of anisotropic a.d.p.s, which were defined with the TLSMD server (http://skuld.bmsc.washington.edu/~tlsmd) (81). Although refinement included standard stereochemistry libraries (82), the interatomic distances involving iron centers were refined without target restraints. The stereochemistry of the refined structures was analyzed using MolProbity (83). A three-dimensional superposition of polypeptide chains was performed using MODELLER (84). All figures were prepared using PyMOL (Schrodinger, 2010). Structure factors and associated structure coordinates of PdG-D-LDH and PdG-D-LDH-lactate complex were deposited in the Protein Data Bank (www.rcsb.org) (85) with PDB codes 9QGZ and 9QIT, respectively.

### Dockings with D-lactate and L-lactate

Docking calculations were performed using AutoDock Vina (86, 87). D-Lactate and L-Lactate were docked on a cuboid docking box centered on the iron ion present in the active site. A total of 10 poses were generated and ranked according to their Vina Scores. The top 1 pose of each substrate was selected for further inspection and analysis.

### Determination of reaction products

Enzymatic pyruvate production was determined in 2 mL of 100 mM phosphate buffer (pH 7.5) containing 10 mM sodium D-lactate and 0, 1, or 10 mM 1,4 BQ, in the absence or presence of 300 U·mL^−1^ catalase (Sigma-Aldrich) from bovine liver (when molecular oxygen was the electron acceptor to reduce hydrogen peroxide to water). The reaction was initiated by adding 5 U·mL^−1^ of functional enzyme PdG-D-LDH at 35°C for 24 h with constant stirring (100 rpm). To monitor the conversion rates of lactate, aliquots (100 µL) of the reaction mixture were collected periodically throughout the incubation time. The reactions were stopped by adding 0.1 mM NaOH to the samples, incubating for 10 min at room temperature, and then neutralizing with 0.1 mM HCl (88). Samples were centrifuged for 4 min at 13,000 rpm, and the supernatants were loaded onto the above-mentioned HPLC system using an Aminex HPX-87H column (Bio-Rad). Elution occurs under isocratic conditions, using a mobile phase of 0.005 N H_2_SO_4_ at a flow rate of 0.6 mL/min at 40°C, as described by (88). The conversion of lactate was detected by absorption at 195 nm. For comparative analysis, calibration curves (0–50 mM) of D-lactate, pyruvate, and acetate were prepared and analyzed under the same conditions. The retention times for D-lactate, pyruvate (Sigma-Aldrich), and acetate (Sigma-Aldrich) were 11.91 ± 0.01 min, 8.04 ± 0.05 min, and 14.21 ± 0.19 min, respectively.

### Product inhibition assays

For inhibition studies, the activity assays were performed in the presence of increasing concentrations of pyruvate, as described in a previously published methodology (89). The reaction mixtures contained 100 mM phosphate buffer, 0.075 mM DCIP, 10 mM D-lactate, and pyruvate concentrations from 0 to 100 mM. The IC₅₀ was calculated from the dose–response curve using non-linear regression with a four-parameter logistic model, which estimates the concentration of inhibitor required to reduce the enzymatic activity by 50%.

## Data Availability

The amino acid sequence of PdG-D-LDH has been deposited to DDBJ under accession number LC901030 and NCBI GenBank under accession number PV774661 and is expected to be accessible after formalization of accessions resumes.

## SUPPLEMENTAL MATERIAL

Supplemental material aem.01480-25-s0001.docx Tables S1 to S10 and Fig. S1 to S13.

ASM does not own the copyrights to Supplemental Material that may be linked to, or accessed through, an article. The authors have granted ASM a non-exclusive, world-wide license to publish the Supplemental Material files. Please contact the corresponding author directly for reuse.
