# Supplementary material for: Shotgun metagenomic mining reveals a new FAD-dependent D-lactate dehydrogenase in an isopod gut microbiome
Source: Appl Environ Microbiol. 2025 Nov 13;91(12):e01480-25. doi: 10.1128/aem.01480-25 (PMC12724367; doi:10.1128/aem.01480-25)
Supplement: Supplemental material — Tables S1 to S10 and Fig. S1 to S13. [file aem.01480-25-s0001.docx]

**SUPPLEMENTARY MATERIAL**

**Shotgun Metagenomic Mining Reveals a New FAD-dependent D-Lactate Dehydrogenase in an Isopod Gut Microbiome**

Catarina Coelho^1,2^, André Taborda^1^, Constança Lorena^1^, Tomás Frazão^1^, António Veríssimo^2^, Patrícia T. Borges^1^, Vânia Brissos^1^, Igor Tiago^2*^, Lígia O. Martins^1*^

*^1^ Instituto de Tecnologia Química e Biológica António Xavier, Universidade Nova de Lisboa, Av. da República, 2780-15 Oeiras, Portugal; ^2^ Centro de Ecologia Funcional, Departamento Ciências da Vida, Universidade de Coimbra, 3000-456 Coimbra, Portugal*

***Corresponding authors**

**TABLE OF CONTENTS**

Table S1. Top 5 of the Blast standalone results. 2

Table S2. The 50 nearest blast results of the amino acid sequence of PdG-D-LDH against sequences deposited in the NCBI database 3

[Table S3. Blast output of the LOX and LDH used in the phylogenetic analysis of PdG-D-LDH. 5](#_Toc203775448)

[Table S4. Biochemical characteristics of the studied D-LDH. 6](#_Toc203775449)

[Table S5. Biochemical characteristics of the studied L-LDH. 7](#_Toc203775450)

[Table S6. Biochemical characteristics of the studied L-LOX. 8](#_Toc203775451)

[Table S7. Apparent kinetic parameters of D-LDHs for D-lactate as electron donor. 9](#_Toc203775452)

[Table S8. Apparent kinetic parameters of L-LDHs for L-lactate as electron donor. 10](#_Toc203775453)

[Table S9. Apparent kinetic constants of L-LOX for L-lactate as electron donor. 11](#_Toc203775454)

[Table S10. Data collection, processing, and refinement statistics for PdG-D-LDH and PdG-D-LDH-lactate. 12](#_Toc203775455)

[Figure S1. Structural superposition of LDHs. 1](#_Toc203775456)3

[Figure S2. Purification, oligomerization states, and FAD distribution of recombinant PdG-D-LDH. 1](#_Toc203775457)4

[Figure S3. Identification of PdGD-LDH cofactor. 1](#_Toc203775458)5

[Figure S4. Enzymatic activity of the PdGD-LDH with different electron acceptors. 1](#_Toc203775459)6

[Figure S5. The thermodynamic stability of PdG-D-LDH. 1](#_Toc203775460)7

[Figure S6. Structural superposition of the two PdG-D-LDH structures. 1](#_Toc203775462)8

[Figure S7. Active site representation of PdG-D-LDH-D-lactate complex structure. 1](#_Toc203775463)9

[Figure S8. Representation of structural domains in the PdG-D-LDH. 20](#_Toc203775464)

[Figure S9. B-factor representation of PdG-D-LDH (9QGZ). 21](#_Toc203775465)

[Figure S10. Active site superposition of of PdGD-LDH and mLDH. 22](#_Toc203775467)

[Figure S11. Bioconversion of D-lactate to pyruvate. 23](#_Toc203775471)

[Figure S12. PdG-D-LDH inhibition by the substrate 1,4-BQ. 24](#_Toc203775472)

[Figure S13. PdG-D-LDH inhibition by the product pyruvate.](#_Toc203775470) 25

Table S1. Top 5 of the Blast standalone results. Selection of candidate sequence from shotgun metagenome data. The five nearest sequences of each query PDB. Only complete sequences without the non-proteinogenic letter “X” were considered in the final selection.

| **query sequence** | **candidate sequence** | **Similarity (%)** | **length** | **mismatches** | **start** | **end** | **e-value** | **bit score** |
| --- | --- | --- | --- | --- | --- | --- | --- | --- |
| **PDB 7QH2** | NODE_10957 | 33.7 | 454 | 284 | 18 | 463 | 5.0E-73 | 237 |
|  | NODE_500 | 33.6 | 459 | 291 | 10 | 463 | 1.3E-72 | 236 |
|  | NODE_151 | 30.4 | 461 | 302 | 11 | 463 | 1.2E-70 | 232 |
|  | NODE_1653 | 35.9 | 414 | 258 | 48 | 460 | 3.7E-70 | 229 |
|  | NODE_6739 | 37.3 | 367 | 220 | 100 | 463 | 1.2E-66 | 218 |
| **PDB 8JDE** | NODE_8179 | 47.2 | 377 | 194 | 16 | 392 | 4.3E-116 | 345 |
|  | NODE_10957 | 33.3 | 465 | 286 | 19 | 471 | 5.9E-69 | 227 |
|  | NODE_1653 | 34.3 | 431 | 262 | 49 | 468 | 1.9E-61 | 207 |
|  | NODE_151 | 29.4 | 469 | 321 | 7 | 471 | 9.1E-53 | 185 |
|  | NODE_500 | 33.1 | 426 | 267 | 53 | 471 | 2.1E-51 | 180 |

**Table S2.** The 50 nearest blast results of amino acid sequence of PdG-D-LDH against sequences deposited in the NCBI database.

| **Description** | **Organism**  **(Scientific Name)** | **Query Cover (%)** | **E value** | **Per. Ident**  **(%)** | **Accession** | |
| --- | --- | --- | --- | --- | --- | --- |
| FAD-binding oxidoreductase | *Nocardiopsis* sp. B62 | 100 | 0.0 | 99.14 | | WP_210840782.1 |
| FAD-binding oxidoreductase | *Nocardiopsis* sp. L17-MgMaSL7 | 100 | 0.0 | 98.28 | | WP_110051400.1 |
| FAD-binding oxidoreductase | *Nocardiopsis* sp. SBT366 | 100 | 0.0 | 98.71 | | WP_049572365.1 |
| FAD-binding oxidoreductase | *Nocardiopsis prasina* | 100 | 0.0 | 98.06 | | WP_017543228.1 |
| FAD-binding protein | *Nocardiopsis eucommiae* | 99 | 0.0 | 96.96 | | QVJ02966.1 |
| FAD-binding oxidoreductase | *Nocardiopsis eucommiae* | 99 | 0.0 | 96.96 | | WP_431869889.1 |
| FAD-binding oxidoreductase | *Nocardiopsis* sp. NPDC058789 | 99 | 0.0 | 96.75 | | WP_378740294.1 |
| FAD-binding oxidoreductase | *Nocardiopsis ganjiahuensis* | 99 | 0.0 | 94.79 | | WP_017586352.1 |
| FAD-binding oxidoreductase | *Nocardiopsis valliformis* | 100 | 0.0 | 93.09 | | WP_017580332.1 |
| FAD-binding oxidoreductase | *Nocardiopsis exhalans* | 99 | 0.0 | 92.62 | | WP_254421781.1 |
| FAD-binding oxidoreductase | *Nocardiopsis metallicus* | 99 | 0.0 | 93.04 | | WP_184363764.1 |
| MULTISPECIES: FAD-binding oxidoreductase | unclassified *Nocardiopsis* | 99 | 0.0 | 94.78 | | WP_357766878.1 |
| FAD-linked oxidase C-terminal domain-containing protein | *Nocardiopsis nanhaiensis* | 98 | 0.0 | 91.47 | | GAB3713084.1 |
| FAD-binding oxidoreductase | *Nocardiopsis terrae* | 98 | 0.0 | 92.79 | | WP_191269930.1 |
| FAD-binding oxidoreductase | *Nocardiopsis listeri* | 98 | 0.0 | 87.34 | | WP_304076559.1 |
| FAD-binding oxidoreductase | *Nocardiopsis listeri* | 98 | 0.0 | 87.12 | | WP_067598491.1 |
| FAD-binding oxidoreductase | *Nocardiopsis* sp. JB363 | 98 | 0.0 | 86.24 | | WP_087101380.1 |
| FAD-binding oxidoreductase | *Nocardiopsis* sp. MG754419 | 98 | 0.0 | 87.96 | | WP_211719392.1 |
| FAD-binding oxidoreductase | Nocardiopsis coralli | 98 | 0.0 | 86 | | WP_193122161.1 |
| FAD-binding oxidoreductase | Nocardiopsis alba | 98 | 0.0 | 88.4 | | WP_357660707.1 |
| FAD-binding oxidoreductase | Nocardiopsis alkaliphila | 98 | 0.0 | 84.25 | | WP_017602706.1 |
| FAD-binding oxidoreductase | Nocardiopsis sp. NPDC006832 | 98 | 0.0 | 86.9 | | WP_358505854.1 |
| FAD-binding oxidoreductase | Nocardiopsis xinjiangensis | 98 | 0.0 | 83.15 | | WP_017607700.1 |
| FAD-binding oxidoreductase | Nocardiopsis alba | 98 | 0.0 | 88.62 | | WP_357222799.1 |
| FAD-binding oxidoreductase | Nocardiopsis alba | 98 | 0.0 | 88.18 | | WP_354897653.1 |
| FAD-binding oxidoreductase | Nocardiopsis codii | 98 | 0.0 | 85.15 | | WP_330090877.1 |
| FAD-binding oxidoreductase | Nocardiopsis alba | 98 | 0.0 | 88.18 | | WP_376721495.1 |
| FAD-binding oxidoreductase | Nocardiopsis alba | 98 | 0.0 | 87.96 | | WP_378689998.1 |
| MULTISPECIES: FAD-binding oxidoreductase | Nocardiopsis | 98 | 0.0 | 88.62 | | WP_017534916.1 |
| FAD-binding oxidoreductase | Nocardiopsis alba | 98 | 0.0 | 88.4 | | WP_357800026.1 |
| FAD-binding oxidoreductase | Nocardiopsis alba | 98 | 0.0 | 88.18 | | WP_355016018.1 |
| FAD-binding oxidoreductase | Streptomonospora nanhaiensis | 98 | 0.0 | 85.59 | | WP_267950155.1 |
| FAD-binding oxidoreductase | Nocardiopsis alba | 98 | 0.0 | 88.4 | | WP_041562000.1 |
| MULTISPECIES: FAD-binding oxidoreductase | Nocardiopsis | 98 | 0.0 | 85.59 | | WP_344175381.1 |
| FAD-binding oxidoreductase | Nocardiopsis alba | 98 | 0.0 | 88.4 | | WP_387608547.1 |
| FAD-binding oxidoreductase | Nocardiopsis alba | 98 | 0.0 | 88.4 | | WP_357723394.1 |
| FAD-binding oxidoreductase | Nocardiopsis alba | 98 | 0.0 | 87.96 | | WP_378768382.1 |
| FAD-binding oxidoreductase | Nocardiopsis sp. CA-288880 | 98 | 0.0 | 84.93 | | WP_433701689.1 |
| FAD-binding oxidoreductase | Nocardiopsis alba | 98 | 0.0 | 88.18 | | WP_358365915.1 |
| FAD linked oxidase, C-terminal domain protein | Nocardiopsis alba ATCC BAA-2165 | 98 | 0.0 | 88.33 | | AFR06073.1 |
| FAD-binding oxidoreductase | Nocardiopsis sp. NPDC058631 | 98 | 0.0 | 84.03 | | WP_378780614.1 |
| FAD-binding oxidoreductase | Nocardiopsis quinghaiensis | 99 | 0.0 | 84.13 | | WP_150245696.1 |
| FAD-binding oxidoreductase | Nocardiopsis tropica | 98 | 0.0 | 84.72 | | WP_344098488.1 |
| FAD-binding oxidoreductase | Nocardiopsis sp. NPDC101807 | 98 | 0.0 | 84.93 | | WP_402910467.1 |
| FAD-binding oxidoreductase | Nocardiopsis sinuspersici | 98 | 0.0 | 84.28 | | WP_179811410.1 |
| FAD-binding oxidoreductase | Nocardiopsis tropica | 98 | 0.0 | 84.72 | | WP_330161375.1 |
| MULTISPECIES: FAD-binding oxidoreductase | Nocardiopsis | 98 | 0.0 | 84.28 | | WP_077690361.1 |
| MULTISPECIES: FAD-binding oxidoreductase | unclassified Nocardiopsis | 98 | 0.0 | 84.68 | | WP_073701130.1 |
| FAD-binding oxidoreductase | Glycomyces fuscus | 98 | 0.0 | 85.81 | | PDP88169.1 |
| FAD-binding oxidoreductase | Nocardiopsis sp. RV163 | 98 | 0.0 | 85.81 | | WP_047867433.1 |

Table S3. Blast output of the LOX and LDH used in the phylogenetic analysis of PdG-D-LDH. NS- no similarity.

| **Enzyme** | **Organism /strain** | **e-value** | **Identity (%)** | **Accession** | **PDB** | **Reference** |
| --- | --- | --- | --- | --- | --- | --- |
| **Enzymes biochemically characterized** | | | | | | |
| D-LDH | *Mus musculus* | 9.00E-67 | 33.3 | 10090 | 8JDE | (1) |
| D-LDH | *Acetobacterium woodii* | 8.00E-76 | 33.7 | WP014355267.1 | 7QH2 | (2) |
| D-LDH | *Acidobacter aceti* | 9.00E-06 | 25.8 | BCK74409.1 | - | (3) |
| D-LDH | *Acidocella sp.* | 7.00E-04 | 26.0 | EKM99009.1 | - | (3) |
| D-LDH | *Gluconobacter oxidans 2071* | 9.00E-08 | 24.8 | AAW61807.1 | - | (4) |
| D-LDH | *Gluconobacter oxidans 1253* | 6.00E-66 | 32.4 | AAW61014.1 | - | (4) |
| D-LDH | *Paracoccus denitrificans* | - | NS | SFR00537 | - | (5) |
| D-LDH | *Corynebacterium glutamicium* |  |  | YP_225194 | - | (6) |
| D-LDH | *Pseudomonas putida* KT2440 | 5.00E-30 | 28.2 | AAN70309.1 | - | (7) |
| D-LDH | *Lactobacillus jensenii* (LDH1) | - | NS | ZP04645201.1 | - | (8) |
| D-LDH | *Lactobacillus jensenii* (LDH2) | - | NS | ZP05557096.1 | - | (8) |
| D-LDH | *Lactobacillus jensenii* (LDH3) | - | NS | ZP05866095.1 | - | (8) |
| D-LDH | *Salinipirillum sp.* LH10-3-1 | - | NS | WLF00856 | - | (9) |
| D-LDH | *Lactobacillus bulgarius* | - | NS | M85224 | 1J49 | (10) |
| D-LDH | *Lactobacillus helveticus* | - | NS | P30901 | 2DLD | (11) |
| D-LDH | *Archaeaglobus fulgidus* | 6.00E-57 | 32.7 | WP010877901.1 | - | (12) |
| D-LDH | *Thermoproteus tenax* | 1.00E-65 | 34.2 | CCC81449 | - | (13) |
| D-LDH | *Candidatus C. subterraneum* | 1.00E-96 | 38.5 | BAJ50932.1 | - | (13) |
| D-LDH | *Aeropyrum pernix* | 1.00E-68 | 34.6 | BAA79452.1 | - | (14) |
| D-LDH | *Sulfolobus tokodaii* | 7.00E-73 | 31.7 | BAB65648.1 | - | (15) |
| D-LDH | *Escherichia coli* |  | 24.4 | AAA60530.1 | 1F0X | (16) |
| D-LDH | *Saccharomyces cerevisiae* | - | NS | NP010843 |  | (17) |
| D-LDH | *Arabidopsis thaliana* | - | NS | Q94AX4 |  | (18) |
| L-LDH | *Litopenaeus vannamei* | - | NS | ROT73072.1 |  | (19) |
| L-LDH | *Pseudomonas stutzeri* | - | NS | ADL63037.1 | - | (20) |
| L-LDH | *Pseudomonas aeruginosa* | - | NS | AAG08157.1 | - | (21) |
| L-LDH | *Saccharomyces cerevisiae* | - | NS | P00175.1 | 1FCB | (22) |
| L-LDH | *Neisseria meningitidis* | - | NS | Q7DDC4 |  | (23) |
| L-LDH | *Hansenula anomala* | - | NS | P09437.2 |  | (24) |
| L-LOX | *Aerococcus viridans* | - | NS | Q44467.1 | 2DU2 | (25) |
| L-LOX | *Chlamydomonas reinhardtii* | - | NS | F8WQN2.1 | - | (26) |
| L-LOX | *Nostoc sp* | - | NS | Q8Z0C8.1 | - | (26) |
| L-LOX | *Pedicoccus acidilactici* |  | NS | E0NE46 | 6RHT | (27) |
| L-LOX | *Lactobacillus lactis subsp. cremoris* | - | NS | BBD75334 | - | (28) |
| L-LOX | *Geotrichum candidum* |  |  | AAB37722 | - | (29) |
| **Annotated as LDH or LOX (NCBI database)** | | | | | | |
| D-LDH | *Shewanella oneidensis* | 2.00E-21 | 24.3 | AAN54582.2 | - | (30) |
| D-LDH | *Campylobacter jejuni* | 8.00E-24 | 21.9 | CAL35682.1 | - | (31) |
| D-LDH | *Lactobacillus delbrueckii* | - | NS | CAA42781.1 | - | (32) |
| D-LDH | *Arabidopsis thaliana* |  | 32.3 | Q94AX4 | - | (18) |
| L-LDH | *Bombyx mori* |  | NS | ABS18410 | - | (33) |
| L-LDH | *Bacillus licheniformis* | - | NS | EQM25125.1 | - | (34) |
| L-LOX | *Streptococcus iniae* | - | NS | CAA68903.1 | - | (35) |
| L-LOX | *Streptococcus pneumoniae* | - | NS | WP000120709.1 | - | (35) |
| L-LOX | *Streptococcus cristatus* | - | NS | ABI54451.2 | - | (36) |
| L- LOX | *Lactobacillus lactis* | - | NS | WP038603448.1 | - | (37) |

Table S4. Biochemical characteristics of the studied D-LDH. MTT: 3-(4,5-dimethyl-2-thiazolyl)-2,5-diphenyltetrazolium bromide; MB: methylene blue, DCIP: 2,6-dichlorophenolindophenol; PMS: Phenazine methosulfate; INT: p- Idonitrotetrazolium violet; DMN: dimethylnaphtoquinone; BQ: 1,4-benzoquinone; NQS: naphthoquinone; FC: ferricyanide; FAD: flavin dinucleotide; NAD:- nicotinamide adenine dinucleotide; CoA: coenzyme-A; ( -): not determined; ND: not detected.

| **Organism/Strain** | **Activity for L-lactate** | **Electron**  **acceptor** | **Cof.** | **T_opt_ (ºC)** | **pH_opt_** | **T_m_ (ºC)** | **t_1/2_**  **(min)** | **Accession no.** | **PDB** | **References** |
| --- | --- | --- | --- | --- | --- | --- | --- | --- | --- | --- |
| *P. dilatatus* metagenome | Yes | DCIP, BQ, NQS and O_2_ | FAD | **55** | **7-9** | **79** | 222 (60ºC) | PV774661 | 9QGZ | This work |
| ***Bacteria*** | | | | | | | | | | |
| **FAD-dependent** | | | | | | | | | | |
| *Acetobacter aceti* | ND | DCIP, MTT, MB, FC, | FAD | 65 | 7 | - | - | BCK74409.1 | - | (3) |
| *Acinetobacter calcoaceticus* | ND | DCIP | FAD | - | 7.7 | - | - | - | - | (38) |
| *Acetobacterium woodi* | Yes | DCIP | FAD | 30 | 7 | - | - | 33952 | 7QH2 | (39) |
| *Acidocella sp.* MX-AZ02 | ND | DCIP, MTT, MB, FC | FAD | 65 | 7 | - | - | EKM99009.1 | - | (3) |
| *Gluconobacter oxydans* 2071 | ND | MTT-PMS, DCIP, O_2_ | FAD | 55 | 8 | - | - | YP_192463.1 | - | (4, 40) |
| *Gluconobacter oxydans* 1253 | Yes, detect. | MTT-PMS, DCIP | FAD | - | - | - | - | YP_191670.1 | - | (4) |
| *Corynebacterium glutamicium* | Yes (<5%) | DCIP | FAD | 45 | 7 | - | - | CAF19608.1 | - | (6) |
| *Campylobacter jejuni* | Yes | DCIP | - | - | - | - | - | - | - | (41) |
| *Desulfovibrio vulgaris* |  | PMS, NTB, FC, DCIP | - | - | 7.5-9 | - | - | - | - | (42) |
| *Escherichia coli* | - | MTT-PMS, DCIP | FAD | - | 8-9 | - | - | AAA60530.1 | 1F0X | (16) |
| *Neisseria meningitidis* | Yes | MTT | - | - | 8 | - | - | - | - | (43) |
| *Shewanella oneidensis* | Yes | MTT-PMS | FAD | - | - | - | - | WP_011071700.1 | - | (44, 45) |
| *Pseudomonas putida* KT2440 | Yes | MTT, CoA; cyto-c | FAD | 65 | 8 | - | - | AAN70308.1 | - | (7) |
| *Peptostreptococcus elsdenii* | Yes | FC, INT, DCIP, cyto-c, O_2_ | FAD | - | 7.9 | - | - | - | - | (46) |
| *Paracoccus denitrificans* | Yes | DCIP | FAD | - | - | - | - | WP_011749202 | - | (5) |
| **NAD-dependent*** | | | | | | | | | | |
| *Salinipirillum* sp*. LH10-3-1* | - | NAD | NAD | 25 | 5 | - | - | OQ845910 | - | (9) |
| *Lactobacillus helveticus* | - | NAD | NAD | 60 | 4 | - | - | UO7604. | 2DLD | (47) |
| *Lactobacillus bugarius* | - | NAD | NAD | - | 7.5 | - | - | P26297 | 1J49 | (11) |
| *Lactobacillus jensenii* (LDH1) | - | NAD | NAD | 40 | 8 | 48.6 | 39.4 (45ºC) | ZP_05866095 | - | (8) |
| *Lactobacillus jensenii* (LDH2) | - | NAD | NAD | 40 | 8 | 45.7 | 47.6 (45ºC) | ZP_05557096 | - | (8) |
| *Lactobacillus jensenii* (LDH3) | - | NAD | NAD | 40 | 7 | 55.7 | 72.2 (45ºC) | ZP_04645201 | - | (8, 48) |
| ***Archaea*** | | | | | | | | | | |
| **FAD-dependent** | | | | | | | | | | |
| *Thermoproteus tenax* | ND | MTT, DCIP, INT | FAD | >80 | 7 | - | - | CCC81449.1 | - | (13) |
| *Candidatus Caldiarchaeum subterraneum* | ND | DCIP, MTT, FC | FAD | 90 | 8 | - | - | BAJ50932.1 | - | (13) |
| *Aeropyrum pernix* | ND | MTT, DCIP, INT | FAD | >80 | 7 | - | - | BAA79452.1 | - | (14) |
| *Sulfolobus todokaii* | ND | DCIP, FC, INT, MTT | FAD | 80 | 8.5 | - | - | BAB65648.1 | - | (15) |
| *Archaeoglobu. fulgidus* | ND | PMS-MTT, DMN | FAD | 90 | 8 | - | 105 (83ºC) | WP_010877901.1 | - | (12) |
| ***Eukarya*** | | | | | | | | | | |
| **FAD-dependent** | | | | | | | | | | |
| *Mus musculus* | ND | DCIP | FAD | - | 7.4 | - | - | 10090 | 8JDE | (1) |
| *Arabidopsis thaliana* | Yes | cyto-c, DCIP | FAD | - | - | - | - | O23240 | - | (18) |
| *Saccharomyces cerevisiae* | - | cyto-c, PMS-DCIP | FAD | - | 7.5-8 | - | - | - | - | (49) |

(*) most NAD-dependent lactate dehydrogenases are described for reducing pyruvate to lactate.

Table S5. Biochemical characteristics of the studied L-LDH. MTT: 3-(4,5-dimethyl-2-thiazolyl)-2,5-diphenyltetrazolium bromide; DCIP: 2,6-dichlorophenolindophenol; BQ: 1,4-benzoquinone; NQS: naphthoquinone; FcPF6: ferrocenium hexafluorophosphate; FC: FC: ferricyanide; FAD: flavin dinucleotide; FMN: flavin mononucleotide; Fe-S: iron-sulphur cluster; PES: phenazine ethosulfate; PMS: Phenazine methosulfate; INT: p- Idonitrotetrazolium violet; NAD:- nicotinamide adenine dinucleotide; ( - ): not determined; ND: not detected.

| **Organism/strain** | **Activity for D-lactate** | **Electron acceptor** | **Cof.** | **T_opt_**  **(ºC)** | **pH_opt_** | **T_m_ (ºC)** | **t_1/2_ (min)** | **Accession no.** | **PDB** | **References** |
| --- | --- | --- | --- | --- | --- | --- | --- | --- | --- | --- |
| *P. dilatatus* metagenome | Yes | DCIP, BQ, NQS and O_2_ | FAD | 55 | 7-9 | 79 | 222(60ºC) | PV774661 | 9QGZ | This work |
| ***Bacteria*** | | | | | | | | | | |
| **FMN-dependent** | | | | | | | | | | |
| *Aggregatibacter actinomycetemcomitans* | ND | MTT, PMS | - | - | - | - | - | AAA02769 | - | (50) |
| *Clostridium acetobutylicum* P262 | Yes | MTT, DCIP | - | - | 7.5 | - | - | - | - | (51) |
| *Pseudomonas stutzeri* A1501 | - | MTT | - | 70 | 11 | - | - | ADL63037.1 | - | (21) |
| *Pseudomonas aeruginosa* PA01(LldA) | - | DCIP | FMN | - | - | - | - | NP_251072.1 | - | (52) |
| *Pseudomonas aeruginosa* PA01(LldD) | - | DCIP | FMN | - | - | - | - | NP_253459.1 | - | (52) |
| *Pseudomonas stutzeri* SDM | ND | MTT, DCIP, PMS | FMN | 55 | 9 | - | - | GU373722 | - | (20, 53) |
| *Lactobacillus johnsonii* | - | DCIP, BQ, FcPF_6_, O_2_ | - | - | 5.0 | - | - | A0A137PML5 | - | (54) |
| *Lactobacillus helsingborgensis* | - | DCIP, BQ, FcPF_6_, O_2_ | - | - | 6.0 | - | - | A0A0F4LW09 | - | (54) |
| *Shigella sp.* | - | DCIP, BQ, FcPF_6_, O_2_ | - | - | 8.0 | - | - | A0A1D3DHR6 | - | (54) |
| *Gilliamella bombicola* | - | DCIP, BQ, FcPF_6_, O_2_ | - | - | 5.5 | - | - | A0A1C4CJ33 | - | (54) |
| *Enterococcus avium* | - | DCIP, BQ, FcPF_6_, O_2_ | - | - | 6.0 | - | - | S0K8K6 | - | (54) |
| *Mycobacterium sp.* | - | DCIP | FMN | 70 | 7.7 | - | 10 (55ºC) | - | - | (55) |
| **NAD-dependent** | | | | | | | | | | |
| *Bacillus coagulans* | - | NAD | NAD | - | - | - | - | - | - | (56) |
| *Thermotoga maritima* | - | NAD | NAD | 55 | 7 | - | 150 (90ºC) | - | - | (57) |
| ***Archaea*** | | | | | | | | | | |
| **FMN-dependent** |  | | | | | | | | | |
| *Sulfolobus tokodaii* | - | DCIP, PMS-INT, PES-INT, cyto-c, FC | FMN | >80 | 6.5 | - | - | - | - | (58) |
| ***Eukarya*** | | | | | | | | | | |
| **FMN-dependent** | | | | | | | | | | |
| *Saccharomyces cerevisiae* | - | FC | FMN | - | - | - | - | P00175.1 | 1FCB | (22) |
| *Saccharomyces cerevisiae* | - | DCIP | FMN | - | - | - | - | - | - | (59, 60) |
| *Hansenula anomala* |  | FC | FMN | - | - | - | - | P09437.2 | - | (61) |
| **NAD-dependent** | | | | | | | | | | |
| *Litopenaeus vannamei* | - | NAD | NAD | 45 | 8 | - | - | AEC12822 | - | (62) |
| *Saduria entomon* (muscle) | - | NAD | NAD | - | 9 | - | - | - | - | (63) |

Table S6. Biochemical characteristics of the studied L-LOX. DCIP: 2,6-dichlorophenolindophenol; BQ: 1,4-benzoquinone; NQS: naphthoquinone; FAD: flavin dinucleotide; FMN: flavin mononucleotide; AR: Amplex Red; 4-APP: 4-aminoantipyrine; ( - ): not determined; ND: not detected.

| **Organism/strain** | **Activity for D-lactate** | **Electron acceptors** | **Cofactor** | **T_opt_** | **pH_opt_** | **T_m_ (ºC)** | **t_1/2_ (min)** | **Accession no.** | **PDB** | **References** |
| --- | --- | --- | --- | --- | --- | --- | --- | --- | --- | --- |
| *P. dilatatus* metagenome | Yes | DCIP, BQ, NQS, O_2_ | FAD | 55 | 7-9 | 79 | 222 (60ºC) | PV774661 | 9QGZ | This work |
| ***Bacteria*** | | | | | | | | | | |
| *Lactobacillus lactis* | - | Oxygen* | FMN | - | - | - | - | WP038603448.1 | - | (37) |
| *Lactobacillus subsp. cremoris* | ND | Oxygen*** | FMN | 30 | 7 | 50 | - | A0A4Y3JPV3 | - | (28) |
| *Streptococcus dysgalactiae subsp. equisimilis* | - | Oxygen** | FMN | - | 9.5 | - | - | A0A1C2C8T5 | - | (64) |
| *Streptococcus anginosus* | - | Oxygen** | FMN | - | 9.5 | - | - | A0A0P0N9S2 | - | (64) |
| *Carnobacterium viridans* | - | Oxygen** | FMN | - | 8.5 | - | - | A0A1H0ZFB3 | - | (64) |
| *Enterococcus hermanniensis* | - | Oxygen** | FMN | - | 7.0 | - | - | A0A1L8TI44 | - | (64) |
| *Carnobacterium inhibens subsp. gilichinskyi* | - | Oxygen** | FMN | - | 7.5 | - | - | U5S7L3 | - | (64) |
| *Marinilactibacillus. piezotolerans* | - | Oxygen** | FMN | - | 8.0 | - | - | A0A1I3VLW0 | - | (64) |
| *Aerococcus sanguinicola* | - | Oxygen** | FMN | - | 7.0 | - | - | A0A109RDN9 | - | (64) |
| *Aerococcus viridans* | - | Oxygen** | FMN | - | - | - | - | Q44467.1 | 2DU2 | (64-66) |
| *Pedicoccus acidilactici* | - | Oxygen*** | FMN | - | 5.5 | - | - | E0NE46 | 6RHT | (27, 54) |
| ***Eukarya*** | | | | | | | | | | |
| *Geotrichum candidum* | ND | Oxygen*** | FMN |  | 8-11 | - | - | AAB37722 | - | (29) |
| *Chlamydomonas reinhardtii* | ND | Oxygen* | FMN |  | - | - | - | F8WQN2.1 | - | (26) |
| *Nostoc sp.* | ND | Oxygen* | FMN |  | - | - | - | Q8Z0C8.1 | - | (26) |

* measuring activity using an oxygraph

**activity using colorimetric assay with horseradish peroxidase (HRP) and Amplex Red

***activity using colorimetric assay with HRP and 4-aminoantipyrine

Table S7. Apparent kinetic parameters of D-LDHs for D-lactate as electron donor. MTT: 3-(4,5-dimethyl-2-thiazolyl)-2,5-diphenyltetrazolium bromide; DCIP -2,6-dichlorophenolindophenol, PMS: Phenazine methosulfate; BQ: 1,4-benzoquinone; Cyto-c: cytochrome-c; FC: ferricyanide; NAD: nicotinamide adenine dinucleotide; (-) not determined. (1) Kinetic parameters were determined using the crude extract.

| **Organism/ strain** | **Electron acceptors** | | ***k_cat_***  **(s^-1^)** | **K_m_**  **(mM)** | ***k*_cat_/K_m_**  **(M^-1^⋅s^-1^)** | **V_max_**  **(U/mg)** | **References** |
| --- | --- | --- | --- | --- | --- | --- | --- |
| *P. dilatatus* metagenome | | DCIP | 8.2 ± 0.3 | 1.0 ± 0.1 | (8 ± 1) × 10^3^ | 9.5 | This work |
|  |  | BQ | 1.4 ± 0.1 | 0.52 ± 0.06 | (2.6 ± 0.4) × 10^3^ | 1.60 ± 0.07 |  |
|  |  | Oxygen | 0.03 ± 0.01 | 0.016 ± 0.004 | (1.9 ± 0.8) × 10^3^ | 0.070 ± 0.007 |  |
| ***Bacteria*** | | | | | | | |
| *Acetobacter aceti* | | DCIP | 157 | 0.9 | 1.62 ×10^5^ | - | (3) |
| *Acinetobacter calcoaceticus* | | DCIP | - | 0.31 ± 0.02 | - | - | (38) |
| *Acidocella sp.* MX-AZ02 | | DCIP | 69 | 1.46 | 4.7 × 10^4^ | - | (3) |
| *Corynebacterium glutamicium*^(1)^ | | DCIP | - | 0.61^(1)^ | - | 73.5 ^(1)^ | (6) |
| *Desulfovibrio vulgaris* | | NTB | - | 0.8 | - | - | (42) |
| *Escherichia coli* | | PMS-MTT | - | 0.6 | - | - | (16) |
| *Gluconobacter oxydans* GOX1253 | | DCIP | - | - | - | 54.23 ± 0.25 | (4, 40) |
| *Gluconobacter.oxydans* GOX2071 | | DCIP | - | - | - | 0.61 ± 0.01 | (4) |
| *Mycobacterium sp.* | | DCIP | - | 0.14 | - | - | (55) |
| *Neisseria meningitidis* | | MTT | - | 0.59 | - | 149 | (43) |
| *Paracoccus denitrificans* | | UBQ | - | 0.034 | - | 3 | (5) |
| *Peptostreptococcus elsdenii* | | FC | - | 26 | - | - | (46) |
| *Pseudomonas. putida* KT2440 | | MTT | 17 ± 1.5 | 0.094 ± 0.011 | 1.76 × 10^5^ | - | (7) |
| *Acetobacterium woodii* | | NAD | - | 3.6 |  | - | (2, 39) |
| *Lactobacillus plantarum* | | NAD | 91.3 ± 0.9 | 2.7 ± 0.3 | (3.4 ± 0.0) × 10^4^ | 149.6 ± 1.5 | (56) |
| *Lactobacillus jensenii* (LDH1) | | NAD | 4.42 | 2.75 | 1.61 × 10^3^ | 96.88 | (8) |
| *Lactobacillus jensenii* (LDH2) | | NAD | 3.65 | 2.58 | 1.41 × 10^3^ | 88.71 | (8) |
| *Lactobacillus jensenii* (LDH3) | | NAD | 0.64 | 0.41 | 1.56 × 10^3^ | 41.91 | (8) |
| *Lactobacillus helveticus* CNR232 | | NAD | - | 68.42 |  | - | (9, 47) |
| *Lactobacillus bulgaricus* | | NAD | 150 | 133 | 1.33 × 10^3^ | - | (9, 11) |
| *Salinispirillum sp.* LH10-3-1 | | NAD |  | 1.71 |  | 197.2 | (9) |
| ***Archaea*** | | | | | | | |
| *Thermoproteus tenax* | | DCIP | - | 1.10 | - | - | (13) |
| *Candidatus Caldiarchaeum subterraneus* | | DCIP | - | 0.605 | - | - | (13) |
| *Aeropyrum pernix* | | DCIP | - | 4 | - | - | (13, 14) |
| *Sulfolobus todokaii* | | DCIP  MTT | -  - | 0.091  0.156 | - |  | (15) |
| *Archaeoglobus fulgidus* | | MTT | - | 0.150 | - | 14 | (12) |
| ***Eukarya*** | | | | | | | |
| *Arabidopsis thaliana* ^(1)^ | | DCIP | 1.22 ± 0.03 | 0.32 ± 0.03 ^(1)^ | 3.8 × 10^3 (1)^ | - | (18) |
| *Saccharomyces cerevisiae* | | cyto-c | - | 0.285 | - | - | (49) |
| *Saduria entomon (muscle)* | | NAD | - | 90.04 | - | 56.81 | (63) |
| *Mus musculus* | | NAD | 1 ± 0.01 | 0.120 ± 0.005 | 8.3 × 10^3^ | 1.22 ± 0.01 | (1) |

Table S8. Apparent kinetic parameters of L-LDHs for L-lactate as electron donor. MTT: 3-(4,5-dimethyl-2-thiazolyl)-2,5-diphenyltetrazolium bromide; DCIP -2,6-dichlorophenolindophenol; BQ: 1,4-benzoquinone; Cyto-c: cytochrome-c; NAD: nicotinamide adenine dinucleotide; ( -): not determined; (1) Kinetic parameters were determined using the crude extracts.

| **Organism/ strain** | | **Electron acceptors** | ***k_cat_***  **(s^-1^)** | **K_m_**  **(mM)** | | ***k*_cat_/K_m_**  **(M^-1^⋅s^-1^)** | | **V_max_**  **(U/mg)** | | **References** |
| --- | --- | --- | --- | --- | --- | --- | --- | --- | --- | --- |
| *P. dilatatus* metagenome | DCIP | | 4.7 ± 0.2 | | 59 ± 5 | | 79 ± 6 | | 5.6 | This work |
|  | BQ | | 0.9 ± 0.2 | | 147 ± 14 | | 6.3 ± 1.4 | | 1.9 ± 0.17 |  |
|  | Oxygen* | | 0.034 ± 0.001 | | 9.5 ± 0.3 | | 3.6 ± 0.3 | | 0.05 ± 0.02 |  |
| ***Bacteria*** | | | | | | | | | | |
| *Pseudomonas aeruginosa* PAO1 (LldA) | DCIP | | - | | 1.1 ± 0.08 | | - | | 0.87 ± 0.04 | (52) |
| *Pseudomonas aeruginosa* PAO1 (LldD) | DCIP | | - | | 0.34 ± 0.01 | | - | | 0.2 ± 0.0 | (52) |
| *Aggregatibacter actinomycetemcomitans* | MTT | | - | | 0.15 ± 0.02 | | - | | - | (50) |
| *Pseudomonas stutzeri* A1501^(1)^ | MTT | | - | | 0.055 ± 0.003 | | - | | - | (21) |
| *Pseudomonas stutzeri* SDM | MTT | | - | | 0.029 ± 0.0006 | | - | | 332.4 ± 5.4 | (20) |
| *Escherichia coli* K-12 | MTT | | - | | 0.12 | | - | | 31 | (67) |
| *Clostridium acetobutylicum* P262^(1)^ | MTT | | - | | 3.50 | | - | | 13 | (51, 68) |
| *Lactobacillus johnsonii* | DCIP | | 47.2 ± 2.0 | | 1.35 ± 0.18 | | 3.50 × 10^4^ | | - | (54) |
| *Lactobacillus helsingborgensis* | DCIP | | 102 ± 8 | | 0.52 ± 0.09 | | 1.96 × 10^5^ | | - | (54) |
| *Shigella sp.* | DCIP | | 107 ± 10 | | 5.67 ± 1.24 | | 1.89 × 10^4^ | | - | (54) |
| *Gilliamella bombicola* | DCIP | | 207 ± 29 | | 16.90 ± 2.50 | | 1.23 × 10^4^ | | - | (54) |
| *Enterococcus avium* | DCIP | | 94 ± 7.8 | | 21.60 ± 2.20 | | 4.35 ×10^3^ | | - | (54) |
| *Pediococcus acidilactici* | DCIP | | 61.8 ± 8.5 | | 235 ± 45 | | 2.60 × 10^2^ | | - | (54) |
| *Bacillus coagulans* | NAD | | 11.3 ± 1.7 | | 5.9 ± 0.2 | | (1.9 ± 0.2) × 10^3^ | | 18.7 ± 2.8 | (56) |
| *Thermotoga maritima* | NAD | | - | | 410 | | - | | - | (57) |
| ***Archaea*** | | | | | | | | | | |
| *Sulfolobus tokodaii* | DCIP | | - | | 0.19 | | - | | - | (58) |
| ***Eukarya*** | | | | | | | | | | |
| *Saccharomyces cerevisiae* | DCIP | | 1.04 ± 0.07 | | 0.52 ± 0.03 | | 2.0 × 10^3^ | | - | (60) |
| *Saccharomyces cerevisiae* | Cyto-c | | 273 ± 6 | | 0.49 ± 0.05 | | 2.1 × 10^7^ | | - | (22) |

*activity using colorimetric assay with HRP and Amplex Red

Table S9. Apparent kinetic constants of L-LOX for L-lactate as electron donor. DCIP -2,6-dichlorophenolindophenol, BQ: 1,4-benzoquinone; Cyto-c: cytochrome-c; FC: ferricyanide; ( - ): not determined.

| **Organism/ strain** | **Electron acceptors** | ***k_cat_***  **(s^-1^)** | **K_m_**  **(mM)** | ***k*_cat_/K_m_**  **(M^-1^⋅s^-1^)** | **V_max_**  **(U/mg)** | **References** |
| --- | --- | --- | --- | --- | --- | --- |
| *P. dilatatus* metagenome | DCIP | 4.7 ± 0.2 | 59 ± 5 | 79 ± 6 | 5.6 | This work |
|  | BQ | 0.9 ± 0.2 | 147 ± 14 | 6.3 ± 1.4 | 1.9 ± 0.17 |  |
|  | Oxygen* | 0.034 ± 0.001 | 9.5 ± 0.3 | 3.6 ± 0.3 | 0.05 ± 0.02 |  |
| ***Bacteria*** | | | | | | |
| *Lactobacillus subsp. cremoris* | Oxygen** | - | 1.0 | - | - | (28) |
| *Streptococcus dysgalactiae subsp. equisimilis* | Oxygen* | 14.06 ± 0.31 | 0.62 ± 0.07 | 23 × 10^3^ | - | (64) |
| *Streptococcus anginosus* | Oxygen* | 6.66 ± 0.46 | 0.95 ± 0.27 | 7 × 10^3^ | - | (64) |
| *Carnobacterium viridans* | Oxygen* | 41.42 ± 0.87 | 0.38 ± 0.04 | 110 × 10^3^ | - | (64) |
| *Enterococcus hermanniensis* | Oxygen* | 52.04 ± 1.64 | 0.92 ± 0.18 | 57 × 10^3^ | - | (64) |
| *C. inhibens subsp. gilichinskyi* | Oxygen* | 25.60 ± 0.87 | 0.58 ± 0.13 | 44 × 10^3^ | - | (64) |
| *Marinilactibacillus piezotolerans* | Oxygen* | 51.26 ± 3.48 | 0.31 ± 0.10 | 168 × 10^3^ | - | (64) |
| *Aerococcus sanguinicola* | Oxygen* | 48.39 ± 1.36 | 2.11 ± 0.21 | 23 × 10^3^ | - | (64) |
| *Aerococcus viridans* | Oxygen* | 46.26 ± 1.20 | 0.33 ± 0.05 | 140 ×10^3^ | - | (64) |
| ***Eukarya*** | | | | | | |
| *Nostoc sp* | Oxygen | - | 0.039 ± 0.007 | - | 12.73 ± 1.55 | (26) |
| *Chlamydomonas reinhardtii* | Oxygen | - | 0.081 ± 0.027 | - | 10.59 ± 0.46 | (26) |
| *Geotrichum candidum* | Oxygen** |  | 3.6 |  | 1.26 | (29) |

*activity using colorimetric assay with HRP and Amplex Red

**activity using colorimetric assay with HRP and 4-aminoantipyrine

Table S10. Data collection, processing, and refinement statistics for PdG-D-LDH and PdG-D-LDH-lactate. Values in parentheses belong to the highest resolution shell.

|  | **PdG-D-LDH** | **PdG-D-LDH-lactate** |
| --- | --- | --- |
| **Data collection** | | |
| Beamline | ID30B (ESRF) | XALOC BL-13 (ALBA) |
| Wavelength (Å) | 0.873128 | 0.979180 |
| Space group | P1 | P2_1_ |
| Unit cell (Å, °) | a = 69.67, b = 103.92,   c = 143.83, α=81.94, β=77.24, γ=75.41 | a = 127.23, b = 109.64,  c = 143.44, β=98.26 |
| Resolution (Å) | 78.15-2.84 (3.23-2.84) | 88.11-3.04 (3.29-3.04) |
| Number of observations | 83138 (4337) | 155634 (8178) |
| Unique reflections | 46387 (2319) | 48050 (2402) |
| Completeness (%) | 89.2 (53.8) | 91.6 (67.2) |
| Multiplicity | 1.8 (1.9) | 3.2 (3.4) |
| CC_1/2_ (%)^a^ | 98.70 (57.10) | 98.50 (80.70) |
| Rmeas (%)^b^ | 14.60 (70.10) | 16.70 (67.70) |
| Rpim (%)^c^ | 10.30 (49.50) | 9.10 (36.30) |
| I/<σ(I)> | 3.70 (1.70) | 3.80 (1.60) |
| Wilson B-factor (Å^2^) | 57.89 | 56.69 |
| V_M_ (Å^3^ Da^-1^) | 2.61 | 2.64 |
| Estimated solvent content (%) | 52.90 | 53.43 |
| **Refinement** | | |
| Rwork (%)^d^ | 20.74 | 20.67 |
| Rfree (%)^d^ | 26.23 | 26.33 |
| RMSD for bond lengths (Å) | 0.004 | 0.002 |
| RMSD for bond angles (°) | 0.887 | 0.464 |
| Average chain B-factor (A^2^) | 57.67, 60.35, 63.45, 71.61, 68.45, 66.79, 70.56, 68.44 | 59.70, 50.61, 66.60, 48.72, 67.42, 50.69, 67.77, 50.02 |
| Ramachandran plot |  |  |
| Residues in favored regions (%) | 86.64 | 97.22 |
| Residues in allowed regions (%) | 10.43 | 2.67 |
| Residues in disallowed regions (%) | 2.93 | 0.11 |
| PDB code | 9QGZ | 9QIT |

^a^ CC _1/2_ = Percentage of correlation between intensities from random half-datasets (69)

^b^ R_meas_ = Σ_hkl_ [N/(N(hkl) -1)]^1/2^ Σ_i_ |I_i_(hkl) - <I(hkl) >|/Σ_hkl_ Σ_i_ I_i_ (hkl), where N(hkl) is the data multiplicity, I_i_(hkl) is the observed intensity, and <I(hkl)> is the average intensity of multiple observations from symmetry-related reflections. It indicates the agreement between symmetry-related observations (70).

^c^ R_p.i.m._ = Σ_hkl_ [1/(N(hkl) -1)]^1/2^ Σ_i_ |I_i_(hkl) - <I(hkl) >|/Σ_hkl_ Σ_i_ I_i_ (hkl), where N(hkl) is the data multiplicity, I_i_(hkl) is the observed intensity, and <I(hkl)> is the average intensity of multiple observations from symmetry-related reflections. It indicates the precision of the final merged and average dataset (71)

^d^ R_work_ refers to the actual working data set used in reﬁnement, while R_free_ refers to a cross-validation set that is not directly used in reﬁnement and is therefore free from reﬁnement bias.

**
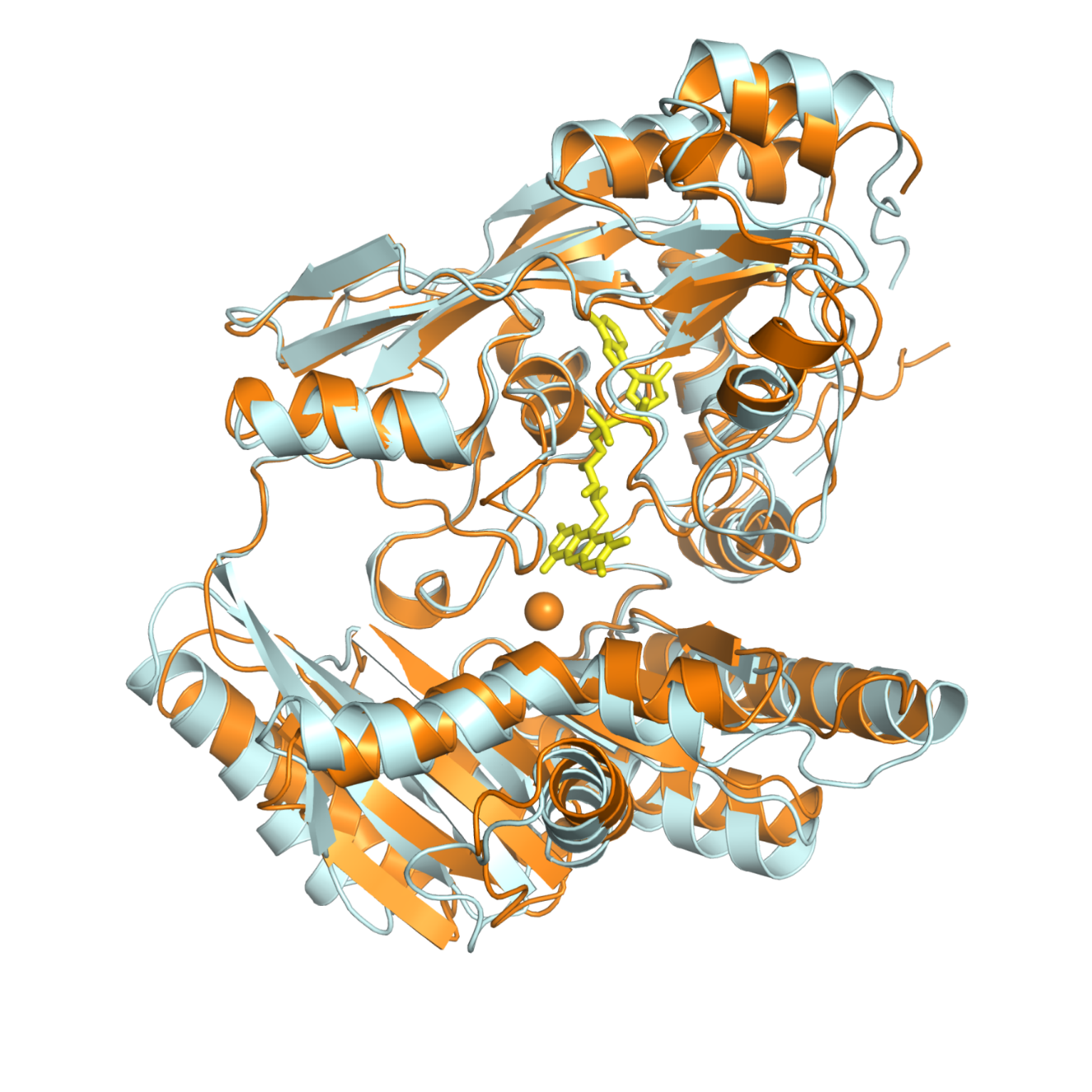
**

Figure S1. Structural superposition of LDHs. Cartoon representation of PdG-D-LDH AlphaFold model (orange) and crystal structure of M. musculus LDH (PDB 8JDE, light blue).

**
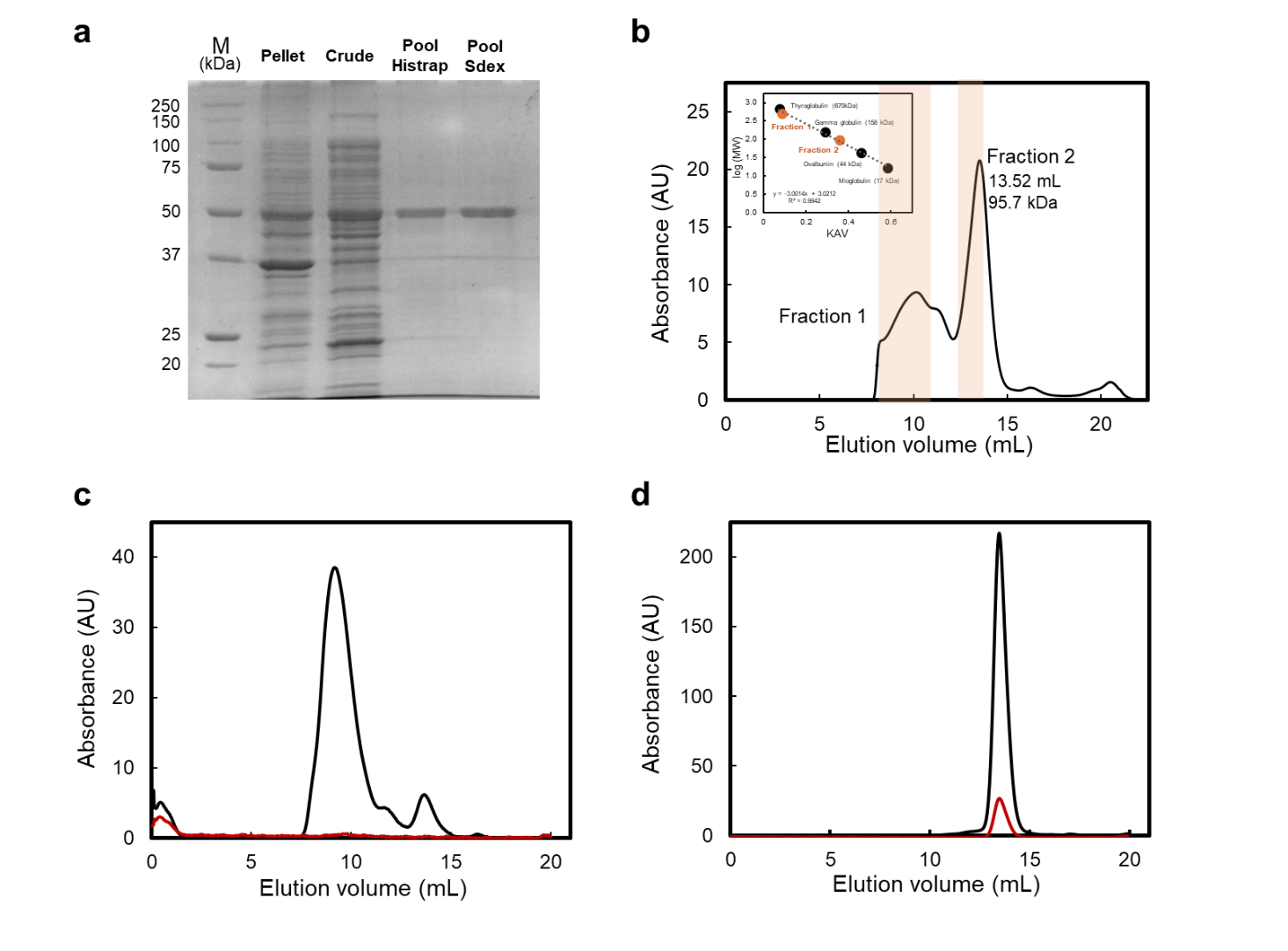
**

Figure S2. Purification, oligomerization states, and FAD distribution of recombinant PdG-D-LDH. (a) SDS-PAGE. M represents the molecular mass marker; the other lanes correspond to the insoluble protein fraction (pellet), the soluble protein fraction (crude extract), the purified enzyme pool obtained from the Histrap column, and the dimeric fraction collected from Superdex 200 (Sdex) column. (b) Size exclusion chromatography chromatogram of PdG D-LDH with two prominent elution peaks. The first peak (fraction 1) at 10 mL corresponds to the molecular mass (439 ± 18 kDa), and the second peak (fraction 2) at 13.52 mL corresponds to the molecular mass (97 ± 2 kDa). The calibration curve was constructed using thyroglobulin (670 kDa), gamma globulin (158 kDa), ovalbumin (44 kDa), and myoglobin (17 kDa) as molecular weight standards to estimate the molecular mass of PdG-D-LDH in solution (minor graph within the principal graph). Size exclusion chromatography analysis of the (c) fraction 1 and (d) fraction 2. The absorbance was measured at 280 nm (black line) and 450 nm (red line).


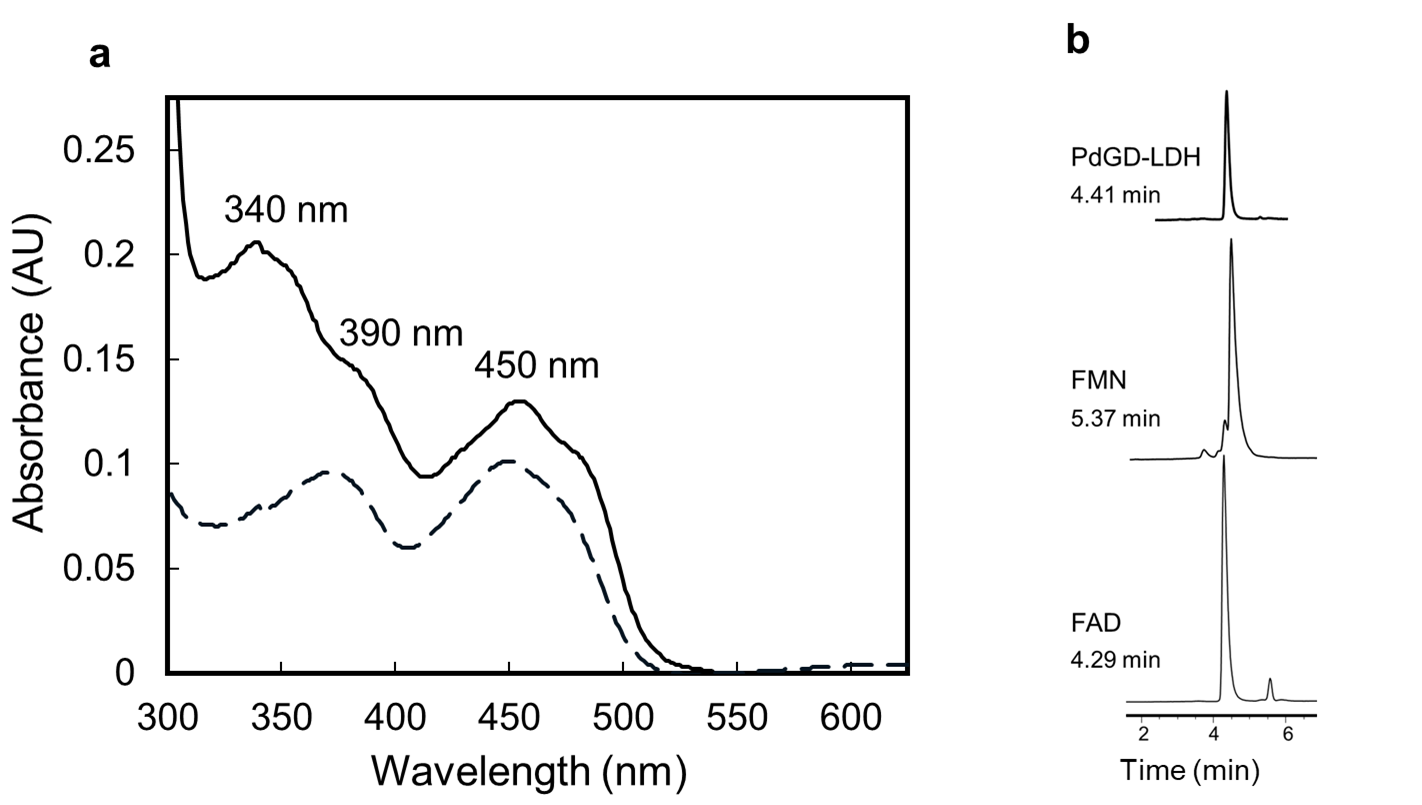


Figure S3. Identification of PdG-D-LDH cofactor. (a) The UV-vis spectrum of PdGD-LDH (black line) and after the denaturation upon adding 0.4 % SDS (grey dashed line). The peak at 340 nm could potentially involve contribution from iron, while the peaks at 390 nm and 450 nm correspond to the typical flavin peaks. (b) HPLC analysis of PdGD-LDH cofactor compared with FAD and FMN standards. The study was performed using two solvent systems: 5 mM ammonium acetate buffer, pH 6.5 (solvent A), and 100% methanol (solvent B), both at 40 ºC. The flavins were detected by absorption at 264 nm.


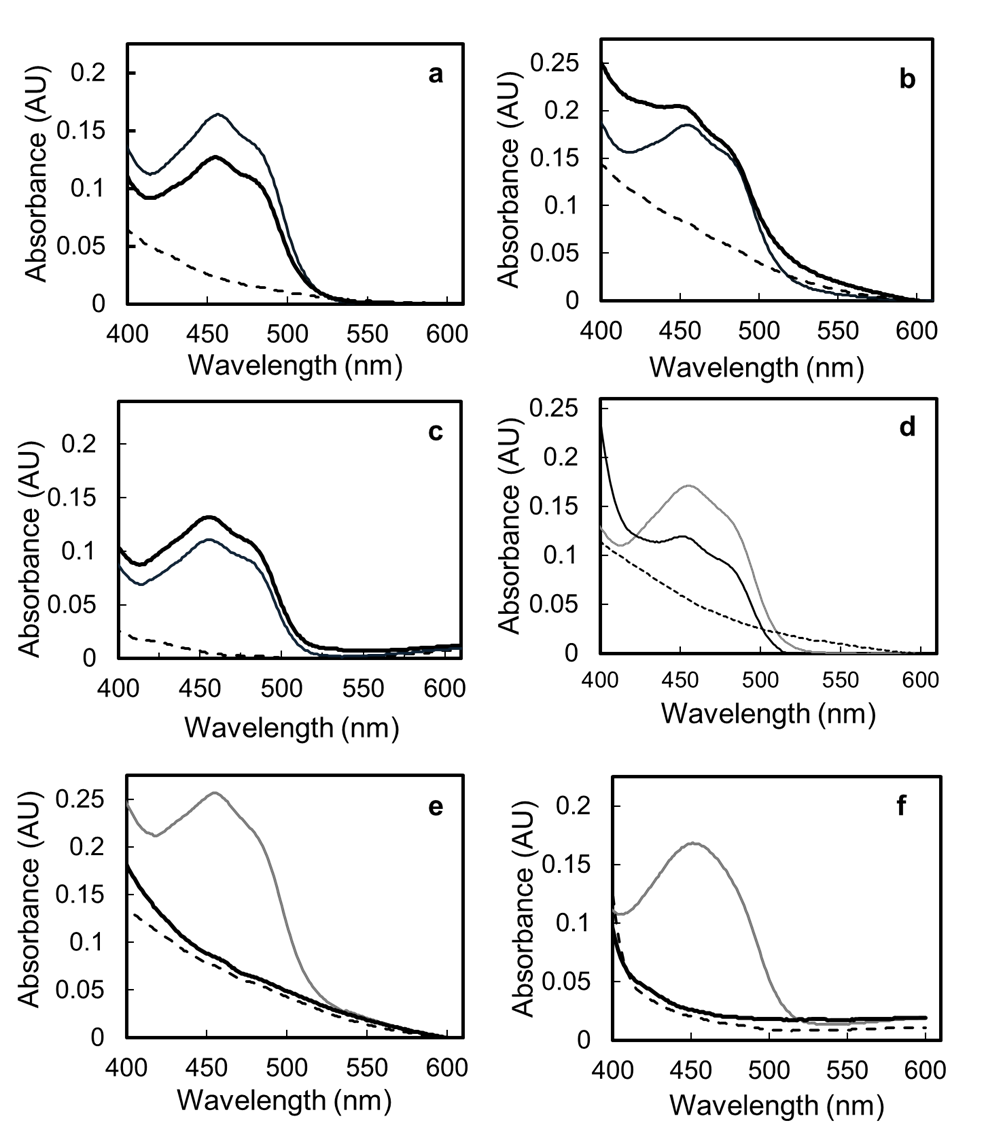


Figure S4. Enzymatic activity of the PdG-D-LDH with different electron acceptors. The native enzyme argon-flushed (grey line) was reduced to 10 mM dithionite (dashed line). Then, each electron acceptor was added separately, and sequential spectra were traced for at least 1 h to follow the typical flavin peak (at 450 nm) (black line). (a) oxygen; (b) DCIP; (c) 1,4-BQ; (d) NQS; (e) NADP; (f) NAD. This procedure was not used with cytochrome c because the reducing agent (dithionite) would also reduce the cytochrome in the reaction mixture, thereby interfering with the assay.

**
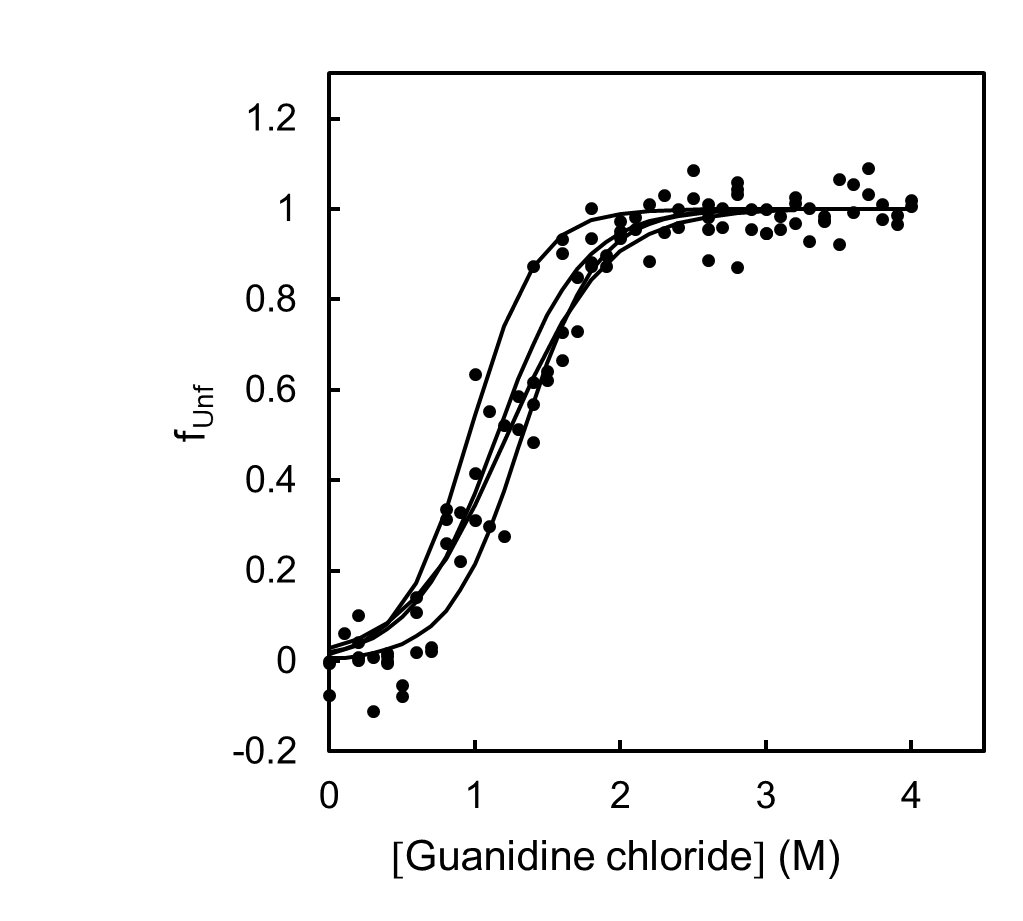
**

Figure S5. The thermodynamic stability of PdG-D-LDH as measured by fluorescence emission during chemical denaturation (GdnHCl). The solid line represents the fit of experimental data through the equation f_U_ = exp(−ΔG°/RT)/ (1 + exp(−ΔG°/RT)). The [GdnHCl]½ was 1.2 M. The free energy change (ΔG) and the m value for GdnHCl-induced unfolding were 2.5 ± 0.4 kcalᐧmol-1 and 2.2 ± 0.4 kcalᐧmol^-1^ᐧM^-1^, respectively.


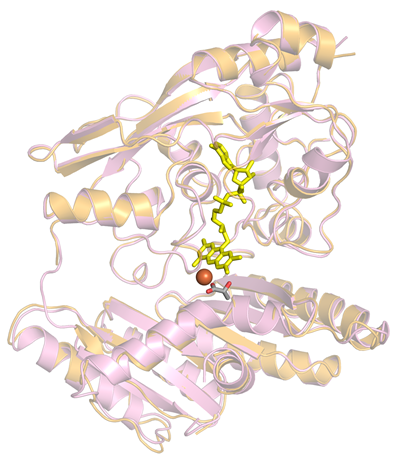


Figure S6. Structural superposition of the two PdG-D-LDH structures. Cartoon representation of PdGD-LDH (orange) and complex with D-lactate (pink). The D-lactate molecule is shown as sticks with gray carbon atoms and red oxygen atoms. The FAD is shown as sticks in yellow. The iron ion is shown as an orange sphere.


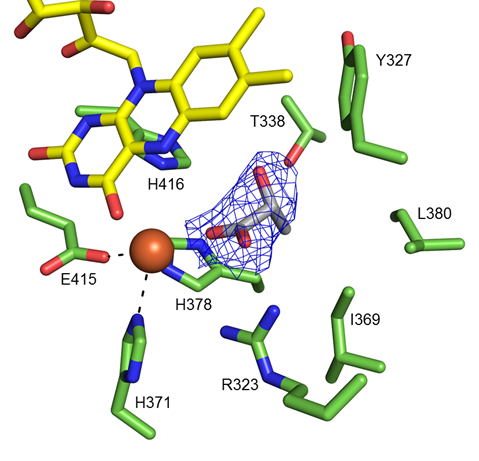


Figure S7. Active site representation of PdG-D-LDH-D-lactate complex structure. Representation of the residues coordinating the iron ion (H371, H378, and E415), and those close to the D-lactate molecule, are shown as sticks with carbon atoms colored green (substrate domain), nitrogen in blue, and oxygen in red. The D-lactate carbon atoms are colored in gray and the oxygen in red. The 2|Fo|- |Fc| map is drawn in blue at the 1.0 r.m.s. level. The bonds established between the iron ion and H371, H378 and E415 are shown as black dashed lines. The FAD is shown as sticks with carbon atoms in yellow, nitrogen in blue, and oxygen in red. The iron ion is shown as an orange sphere.


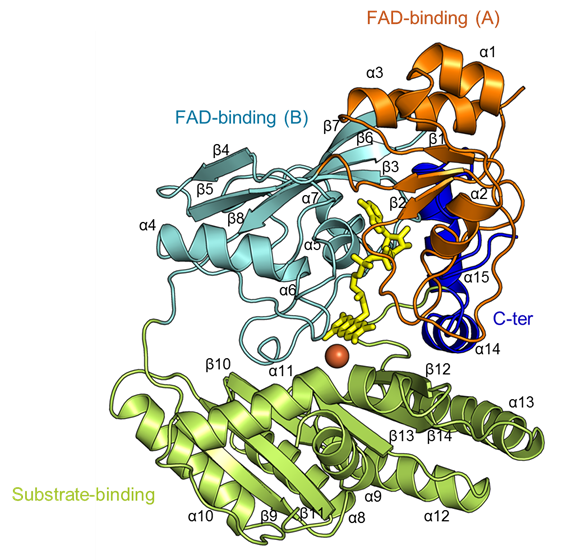


Figure S8. Representation of structural domains in the PdG-D-LDH. Cartoon representation of the monomer with domains colored as in Fig. 4a. The secondary structural elements are labeled. The FAD-binding domain A contains a three-stranded parallel β-sheet (β1–β3) surrounded by three α-helices (α1–α3), and FAD-binding domain B comprises a five-stranded antiparallel β-sheet (β4–β8) surrounded by four α-helices (α4-α7). The substrate-binding domain consists of a six-stranded antiparallel β-sheet (β9–β14) enclosed by six α-helices (α8–α13). The C-terminal domain comprises two α-helices (α14–α15). The FAD is shown as sticks in yellow. The iron ion is shown as an orange sphere.


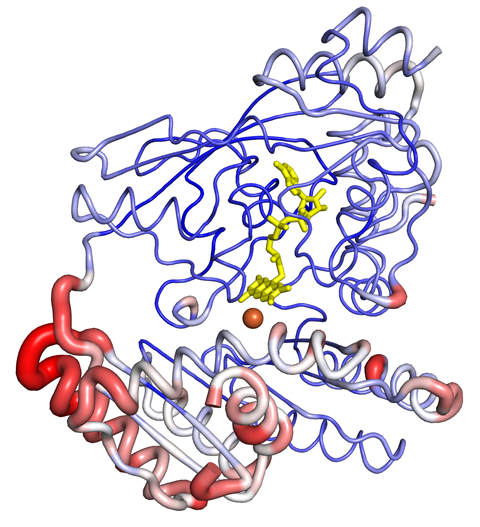


Figure S9. B-factor representation of PdG-D-LDH (9QGZ). Cartoon representation of the main-chain with thickness proportional to <a.d.p.> values, color-coded from blue (more rigid) to red (more flexible).


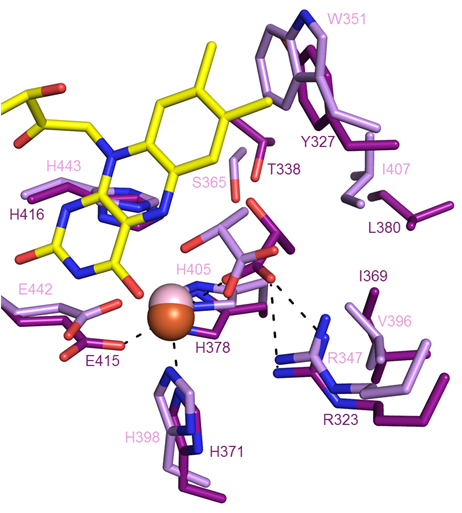


Figure S10. Active site superposition of PdGD-LDH and mLDH. The residues surrounding the substrate binding site in PdGD-LDH and mLDH (*Mus musculus* LDH) are shown as sticks colored in dark purple and light purple, respectively.


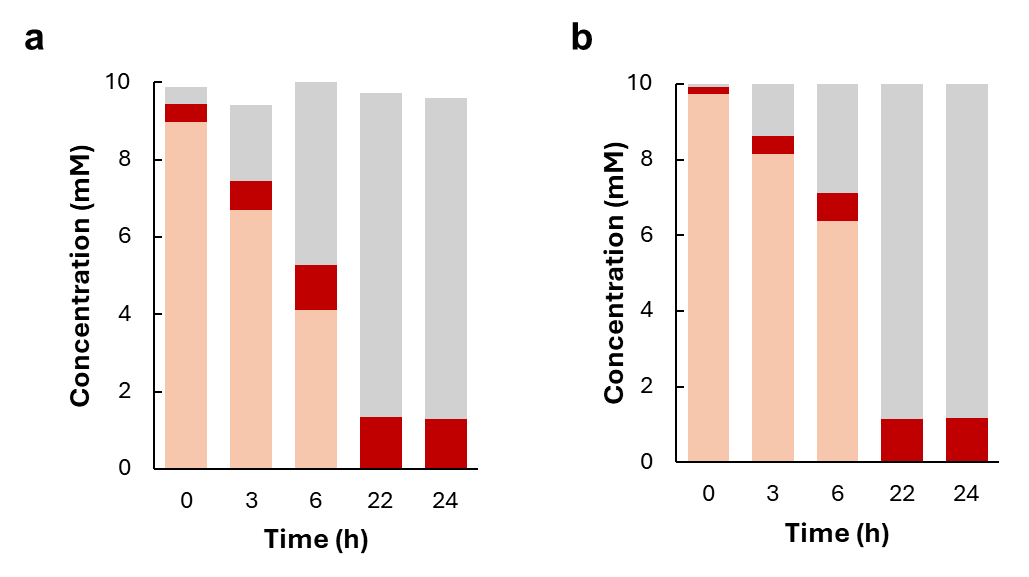


Figure S11. Bioconversion of D-lactate to pyruvate. The bioconversion of 10 mM D-lactate with 1 U/mL enzyme at 25 ºC using as electron acceptor (a) 10 mM 1,4-BQ or (b) oxygen. After 24 h, the 10 mM of D-lactate (pink) were converted into pyruvate (red) and acetate (grey).


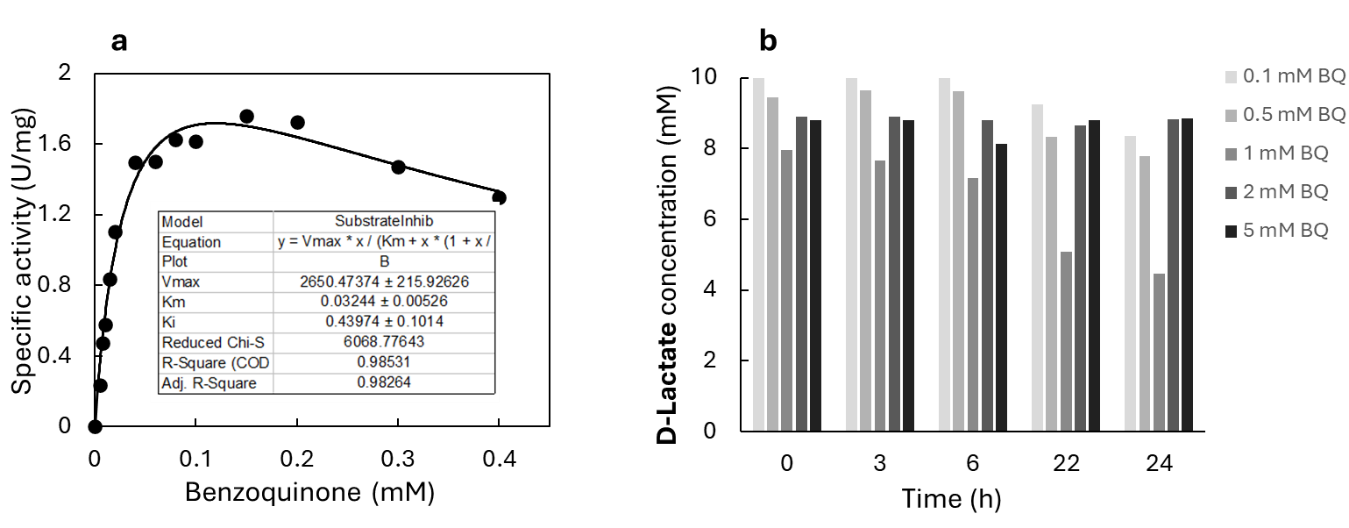


Figure S12. Inhibition of PdG-D-LDH by 1,4-BQ at concentrations higher than 0.1 mM. Kinetic analysis of PdG-D-LDH for the oxidation of 10 mM D-lactate at increasing concentrations of 1,4-BQ as electron acceptor. The steady state kinetic constants were obtained following the equation v=*V*_max_/(1+*K*_m_[S]+[S]/*K*_i_).

**
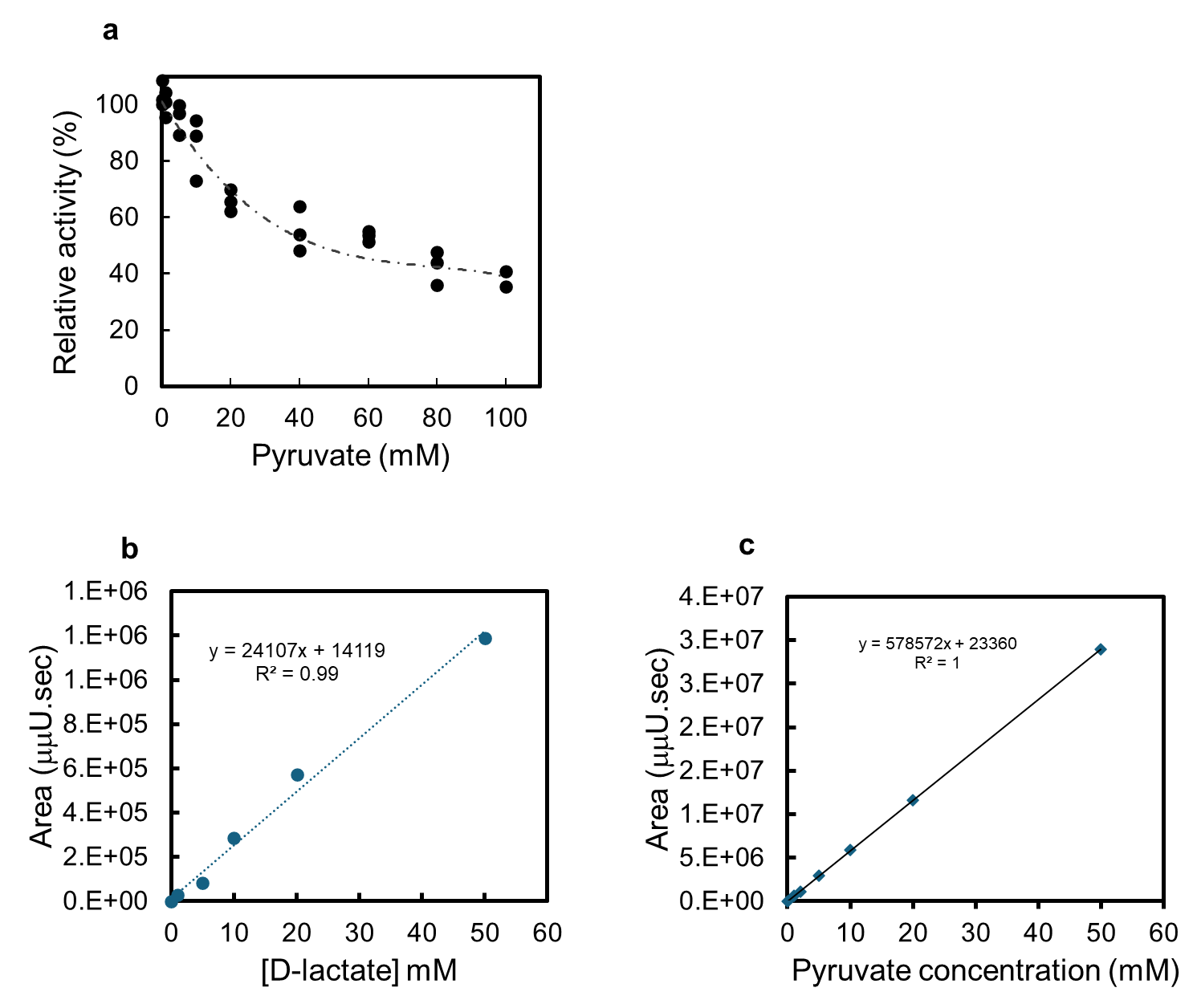
**

Figure S13. PdG-D-LDH inhibition by pyruvate concentration. Pyruvate inhibition was assessed by measuring enzyme activity in the presence of increasing pyruvate concentrations, yielding an IC50 of 53 mM. The reactions, followed by HPLC, were performed using 0.075 mM DCIP, 10 mM D-lactate, and 0-100 mM pyruvate in sodium phosphate buffer (pH 7.5) at room temperature. The IC₅₀ was calculated from the dose–response curve using nonlinear regression with a four-parameter logistic model, which estimates the concentration of inhibitor required to reduce the enzymatic activity by 50%, as depicted in the graph.

**References**

1. Jin S, Chen X, Yang J, Ding J. 2023. Lactate dehydrogenase D is a general dehydrogenase for D-2-hydroxyacids and is associated with D-lactic acidosis. Nat Commun 14:6638.

2. Kayastha K, Katsyv A, Himmrich C, Welsch S, Schuller JM, Ermler U, Müller V. 2022. Structure-based electron-confurcation mechanism of the Ldh-EtfAB complex. eLife 11:e77095.

3. Min K, Yeon YJ, Um Y, Kim YH. 2016. Novel NAD-independent d-lactate dehydrogenases from *Acetobacter aceti* and *Acidocella species* MX-AZ02 as potential candidates for in vitro biocatalytic pyruvate production. Biochem Eng J 105:358-363.

4. Sheng B, Xu J, Zhang Y, Jiang T, Deng S, Kong J, Gao C, Ma C, Xu P. 2015. Utilization of D-lactate as an energy source supports the growth of *Gluconobacter oxydans*. Appl Environ Microbiol 81:4098-4110.

5. Kim G, Covian R, Edwards L, He Y, Balaban RS, Levine RL. 2024. Lactate oxidation in *Paracoccus denitrificans*. Arch Biochem Biophys 756:109988.

6. Kato O, Youn JW, Stansen KC, Matsui D, Oikawa T, Wendisch VF. 2010. Quinone-dependent D-lactate dehydrogenase Dld (Cg1027) is essential for growth of *Corynebacterium glutamicum* on D-lactate. BMC Microbiol 10:321-321.

7. Jiang T, Guo X, Yan J, Zhang Y, Wang Y, Zhang M, Sheng B, Ma C, Xu P, Gao C. 2017. A bacterial multidomain NAD-independent D-lactate dehydrogenase utilizes flavin adenine dinucleotide and Fe-S clusters as cofactors and quinone as an electron acceptor for D-lactate oxidization. J Bacteriol 199:e00342-17.

8. Jun C, Sa YS, Gu SA, Joo JC, Kim S, Kim KJ, Kim YH. 2013. Discovery and characterization of a thermostable D-lactate dehydrogenase from *Lactobacillus jensenii* through genome mining. Process Biochem 48:109-117.

9. Liu J, Jiang X, Zheng Y, Li K, Zhang R, Xu J, Wang Z, Zhang Y, Yin H, Li J. 2024. Expression, characterization, and immobilization of a novel D-lactate dehydrogenase from *Salinispirillum* sp. LH 10-3-1. Processes 12:1349.

10. Razeto A, Kochhar S, Hottinger H, Dauter M, Wilson KS, Lamzin VS. 2002. Domain closure, substrate specificity and catalysis of D-lactate dehydrogenase from *Lactobacillus bulgaricus*. J Mol Biol 318:109-119.

11. Kochhar S, Hunziker PE, Leong-Morgenthaler P, Hottinger H. 1992. Primary structure, physicochemical properties, and chemical modification of NAD+-dependent D-lactate dehydrogenase. Evidence for the presence of ARG- 235, HIS-303, TYR-101, and TRP-19 at or near the active site. J Biol Chem 267:8499-8513.

12. Reed DW, Hartzell PL. 1999. The *Archaeoglobus fulgidus* D-lactate dehydrogenase is a Zn(2+) flavoprotein. J Bacteriol 181:7580-7587.

13. Satomura T, Hayashi J, Sakamoto H, Nunoura T, Takaki Y, Takai K, Takami H, Ohshima T, Sakuraba H, Suye Si. 2018. D-Lactate electrochemical biosensor prepared by immobilization of thermostable dye-linked D-lactate dehydrogenase from *Candidatus Caldiarchaeum subterraneum*. J Biosci Bioeng 126:425-430.

14. Shibahara T, Satomura T, Kawakami R, Ohshima T, Sakuraba H. 2011. Crystallization and preliminary X-ray analysis of a dye-linked D-lactate dehydrogenase from the aerobic hyperthermophilic archaeon *Aeropyrum pernix*. Acta Crystallogr Sect F 67:1425-1427.

15. Satomura T, Kawakami R, Sakuraba H, Ohshima T. 2008. A novel flavin adenine dinucleotide (FAD) containing d-lactate dehydrogenase from the thermoacidophilic crenarchaeota *Sulfolobus tokodaii* strain 7: Purification, characterization and expression in *Escherichia coli*. J Biosci Bioeng 106:16-21.

16. Futai M. 1973. Membrane D-lactate dehydrogenase from *Escherichia coli.* Purification and properties. Biochem 12:2468-2474.

17. Becker-Kettern J, Paczia N, Conrotte JF, Kay DP, Guignard C, Jung PP, Linster CL. 2016. *Saccharomyces cerevisiae* forms D-2-hydroxyglutarate and couples its degradation to D-lactate formation via a cytosolic transhydrogenase. J Biol Chem 291:6036-58.

18. Engqvist M, Drincovich MF, Flügge UI, Maurino VG. 2009. Two D-2-hydroxy-acid dehydrogenases in arabidopsis thaliana with catalytic capacities to participate in the last reactions of the methylglyoxal and β-oxidation pathways. J Biol Chem 284:25026-25037.

19. Fregoso-Peñuñuri AA, Valenzuela-Soto EM, Figueroa-Soto CG, Peregrino-Uriarte AB, Ochoa-Valdez M, Leyva-Carrillo L, Yepiz-Plascencia G. 2017. White shrimp *Litopenaeus vannamei* recombinant lactate dehydrogenase: Biochemical and kinetic characterization. Protein Expr Purif 137:20-25.

20. Gao C, Jiang T, Dou P, Ma C, Li L, Kong J, Xu P. 2012. NAD-independent L-lactate dehydrogenase is required for L-lactate utilization in *Pseudomonas stutzeri* SDM. PLoS ONE 7:5.

21. Gao C, Wang Y, Zhang Y, Lv M, Dou P, Xu P, Ma C. 2015. NAD-independent L-lactate dehydrogenase required for L-lactate utilization in *Pseudomonas stutzeri* A1501. J Bacteriol 197:2239-2247.

22. Cunane LM, Barton JD, Chen ZW, Welsh FE, Chapman SK, Reid GA, Mathews FS. 2002. Crystallographic study of the recombinant flavin-binding domain of baker's yeast flavocytochrome b2: Comparison with the intact wild-type enzyme. Biochemistry 41:4264-4272.

23. Erwin AL, Gotschlich EC. 1996. Cloning of a *Neisseria meningitidis* gene for L-lactate dehydrogenase (L- LDH): Evidence for a second meningococcal L-LDH with different regulation. J Bacteriol 178:4807-4813.

24. Dong JM, Taylor JS, Latour DJ, Iuchi S, Lin ECC. 1993. Three overlapping lct genes involved in L-lactate utilization by Escherichia coli. Journal of Bacteriology 175:6671-6678.

25. Duncan JD, Wallis JO, Azari MR. 1989. Purification and properties of *Aerococcus viridans* lactate oxidase. BBRC 164:919-926.

26. Hackenberg C, Kern R, Hüge J, Stal LJ, Tsuji Y, Kopka J, Shiraiwa Y, Bauwe H, Hagemann M. 2011. Cyanobacterial lactate oxidases serve as essential partners in N2 fixation and evolved into photorespiratory glycolate oxidases in plants. Plant Cell 23:2978-2990.

27. Ashok Y, Maksimainen MM, Kallio T, Kilpeläinen P, Lehtiö L. 2020. FMN-dependent oligomerization of putative lactate oxidase from *Pediococcus acidilactici*. PLoS ONE 15:2.

28. Toda A, Nishiya Y. 1998. Gene Cloning, Purification, and Characterization of a Lactate Oxidase from Lactococcus Zactis subsp. cremoris IF03427. J Ferment Bioeng 85:507-510.

29. Sztajer H, Wang W, Lu H, Stocker A, Schmid RD. 1996. Purification and some properties of a novel microbial lactate oxidase. Appl Microbiol Biotechnol 45:600-606.

30. Heidelberg JF, Paulsen IT, Nelson KE, Gaidos EJ, Nelson WC, Read TD, Eisen JA, Seshadri R, Ward N, Methe B, Clayton RA, Meyer T, Tsapin A, Scott J, Beanan M, Brinkac L, Daugherty S, DeBoy RT, Dodson RJ, Durkin AS, Haft DH, Kolonay JF, Madupu R, Peterson JD, Umayam LA, White O, Wolf AM, Vamathevan J, Weidman J, Impraim M, Lee K, Berry K, Lee C, Mueller J, Khouri H, Gill J, Utterback TR, McDonald LA, Feldblyum TV, Smith HO, Venter JC, Nealson KH, Fraser CM. 2002. Genome sequence of the dissimilatory metal ion-reducing bacterium *Shewanella oneidensis*. Nat Biotechnol 20:1118-1123.

31. Parkhill J, Wren BW, Mungall K, Ketley JM, Churcher C, Basham D, Chillingworth T, Davies RM, Feltwell T, Holroyd S, Jagels K, Karlyshev AV, Moule S, Pallen MJ, Penn CW, Quail MA, Rajandream MA, Rutherford KM, van Vliet AH, Whitehead S, Barrell BG. 2000. The genome sequence of the food-borne pathogen *Campylobacter jejuni* reveals hypervariable sequences. Nature 403:665-668.

32. Bernard N, Ferain T, Garmyn D, Hols P, Delcour J. 1991. Cloning of the D-lactate dehydrogenase gene from *Lactobacillus delbrueckii* subsp. *bulgaricus* by complementation in *Escherichia coli*. FEBS Lett 290:61-64.

33. Zhu M, Chen K, Yao Q. 2007. Identification and characterization of *Bombyx mori* LDH gene through bioinformatics approaches. Int J Indust Entomol 15:137-143.

34. Gálvez EJC, Carrillo-Castro K, Zárate L, Güiza L, Pieper DH, García-Bonilla E, Salazar M, Junca H. 2016. Draft genome sequence of *Bacillus licheniformis* CG-B52, a highly virulent bacterium of Pacific white shrimp (*Litopenaeus vannamei*), isolated from a Colombian Caribbean aquaculture outbreak. Genome Announc 4:e00321-16.

35. Gibello A, Collins MD, Domínguez L, Fernández-Garayzábal JF, Richardson PT. 1999. Cloning and analysis of the L-lactate utilization genes from *Streptococcus iniae*. Appl Environ Microbiol 65:4346-4350.

36. Tong HC, Chen W, Merritt J, Qi FX, Shi WY, Dong XZ. 2007. *Streptococcus oligofermentans* inhibits *Streptococcus mutans* through conversion of lactic acid into inhibitory H_2_O_2_: a possible counteroffensive strategy for interspecies competition. Mol Microbiol 63:872-880.

37. Barré O, Mourlane F, Solioz M. 2007. Copper induction of lactate oxidase of *Lactococcus lactis:* A novel metal stress response. J Bacteriol 189:5947-5954.

38. Allison N, O'Donnell MJ, Fewson CA. 1985. Membrane-bound lactate dehydrogenases and mandelate dehydrogenases of *Acinetobacter calcoaceticus*. Location and regulation of expression. Biochem J 227:753-757.

39. Weghoff MC, Bertsch J, Müller V. 2015. A novel mode of lactate metabolism in strictly anaerobic bacteria. Environ Microbiol 17:670-677.

40. Sheng B, Xu J, Ge Y, Zhang S, Wang D, Gao C, Ma C, Xu P. 2016. Enzymatic resolution by a D-lactate oxidase catalyzed reaction for (S)-2-hydroxycarboxylic acids. ChemCatChem 8:2630-2633.

41. Thomas MT, Shepherd M, Poole RK, Van Vliet AHM, Kelly DJ, Pearson BM. 2011. Two respiratory enzyme systems in *Campylobacter jejuni* NCTC 11168 contribute to growth on L-lactate. Environ Microbiol 13:48-61.

42. Ogata M, Arihara K, Yagi T. 1981. D-lactate dehydrogenase of *Desulfovibrio vulgaris*. J Biochem 89:1423-1431.

43. Erwin AL, Gotschlich EC. 1993. Oxidation of D-lactate and L-lactate by *Neisseria meningitidis*: Purification and cloning of meningococcal D-lactate dehydrogenase. J Bacteriol 175:6382-6391.

44. Brutinel ED, Gralnick JA. 2012. Preferential utilization of d-Lactate by *Shewanella oneidensis*. Appl Environ Microbiol 78:8474-8476.

45. Pinchuk GE, Rodionov DA, Yang C, Li X, Osterman AL, Dervyn E, Geydebrekht OV, Reed SB, Romine MF, Collart FR, Scott JH, Fredrickson JK, Beliaev AS. 2009. Genomic reconstruction of *Shewanella oneidensis* MR-1 metabolism reveals a previously uncharacterized machinery for lactate utilization. Proc Nat Acad Sci USA 106:2874-2879.

46. Brockman HL, Wood WA. 1975. D-Lactate dehydrogenase of *Peptostreptococcus elsdenii*. J Bacteriol 124:1454-61.

47. Bhowmik T, Lueck M, Steele JL. 1993. Purification and partial characterization of D-(-)-lactate dehydrogenase from *Lactobacillus helveticus* CNRZ 32. J Ind Microbiol Biotechnol 12:35-41.

48. Kim S, Gu SA, Kim YH, Kim KJ. 2014. Crystal structure and thermodynamic properties of d-lactate dehydrogenase from *Lactobacillus jensenii*. Int J Biol Macromol 68:151-157.

49. Gregolin C, Singer TP. 1963. The lactic dehydrogenase of yeast: III. d(−)lactic cytochrome c reductase, a zinc-flavoprotein from aerobic yeast. BBA 67:201-218.

50. Brown SA, Whiteley M. 2009. Characterization of the L-lactate dehydrogenase from *Aggregatibacter actinomycetemcomitans*. PLoS ONE 4:11.

51. Diez-Gonzalez F, Russell JB, Hunter JB. 1997. NAD-independent lactate and butyryl-CoA dehydrogenases of *Clostridium acetobutylicum* P262. Curr Microbiol 34:162-166.

52. Wang Y, Xiao D, Liu Q, Zhang Y, Hu C, Sun J, Yang C, Xu P, Ma C, Gao C. 2018. Two NAD-independent L-lactate dehydrogenases drive L-lactate utilization in *Pseudomonas aeruginosa* PAO1. Environ Microbiol Rep 10:569-575.

53. Jiang T, Gao C, Dou P, Ma C, Kong J, Xu P. 2012. Rationally re-designed mutation of NAD-independent L-lactate dehydrogenase: high optical resolution of racemic mandelic acid by the engineered *Escherichia coli*. Microb Cell Fact 11:151.

54. Tsvik L, Steiner B, Herzog P, Haltrich D, Sützl L. 2022. Flavin mononucleotide-dependent L-lactate dehydrogenases: Expanding the toolbox of enzymes for L-lactate biosensors. ACS Omega 7:41480-41492.

55. Xu P, Yano T, Yamamoto K, Suzuki H, Kumagai H. 1996. Characterization of a lactate oxidase from a strain of gram negative bacterium from soil. Appl Biochem Biotechnol 56:277-288.

56. Wang L, Cai Y, Zhu L, Guo H, Yu B. 2014. Major role of NAD-dependent lactate dehydrogenases in the production of L-lactic acid with high optical purity by the thermophile *Bacillus coagulans*. Appl Environ Microbiol 80:7134-7141.

57. Wrba A, Jaenicke R, Huber R, Stetter KO. 1990. Lactate dehydrogenase from the extreme thermophile *Thermotoga maritima*. Eur J Biochem 188:195-201.

58. Satomura T, Uno K, Kurosawa N, Sakuraba H, Ohshima T, Suye SI. 2021. Characterization of a novel thermostable dye-linked L-lactate dehydrogenase complex and its application in electrochemical detection. Int J Mol Sci 22:24.

59. Black MT, Gunn FJ, Chapmant SK, Reid GA. 1989. Structural basis for the kinetic differences between flavocytochromes b2 from the yeasts *Hansenula anomala* and *Saccharomyces cerevisiae*. Biochem J 263:973-976.

60. Boyarski A, Shlush N, Paz S, Eichler J, Alfonta L. 2023. Electrochemical characterization of a dual cytochrome-containing lactate dehydrogenase. Bioelectrochem 152.

61. Gervais M, Risler Y, Corazzin S. 1983. Proteolytic cleavage of *Hansenula anomala* Flavocytochrome b2 into its two functional domains: Isolation of a highly active flavodehydrogenase and a cytochrome b2 core. Eur J Biochem 130:253-259.

62. Leyva-Carrillo L, Hernandez-Palomares M, Valenzuela-Soto EM, Figueroa-Soto CG, Yepiz-Plascencia G. 2019. Purification and partial biochemical characterization of recombinant lactate dehydrogenase 1 (LDH-1) of the white shrimp *Litopenaeus vannamei*. Protein Expr Purif 164:105461.

63. Mulkiewicz E, Stachowiak K, Skorkowski EF. 2000. Properties of lactate dehydrogenase from the isopod, *Saduria entomon*. Comp Biochem Physiol B Biochem Mol Biol 126:337-346.

64. Tsvik L, Zhang S, O’Hare D, Haltrich D, Sützl L. 2024. More than one enzyme: Exploring alternative FMN-dependent L-lactate oxidases for biosensor development. ACS Omega 9:29442-29452.

65. Morimoto Y, Yorita K, Aki K, Misakf H, Massey V. 1998. L-lactate oxidase from *Aerococcus viridans* crystaRized as an octamer. Preliminary X-ray studies. Biochimie 80:309-312.

66. Umena Y, Yorita K, Matsuoka T, Kita A, Fukui K, Morimoto Y. 2006. The crystal structure of l-lactate oxidase from *Aerococcus viridans* at 2.1 Å resolution reveals the mechanism of strict substrate recognition. BBRC 350:249-256.

67. Futai M, Kimura H. 1977. Inducible membrane bound L lactate dehydrogenase from *Escherichia coli*. Purification and properties. J Biol Chem 252:5820-5827.

68. Diez-Gonzalez F, Russell JB, Hunter JB. 1995. The role of an NAD-independent lactate dehydrogenase and acetate in the utilization of lactate by *Clostridium acetobutylicum* strain P262. Arch Microbiol 164:36-42.

69. Karplus PA, Diederichs K. 2012. Linking Crystallographic Model and Data Quality. Science 336:1030-1033.

70. Diederichs K, Karplus PA. 1997. Improved R-factors for diffraction data analysis in macromolecular crystallography. Nat Struct Biol 4:269-275.

71. Weiss MS. 2001. Global indicators of X-ray data quality. J Appl Crystallogr 34:130-135.
